# Costs of Transfer From Nontrauma to Trauma Centers Among Patients With Minor Injuries

**DOI:** 10.1001/jamanetworkopen.2024.34172

**Published:** 2024-09-20

**Authors:** Bourke W. Tillmann, Avery B. Nathens, Matthew P. Guttman, Priscila Pequeno, Damon C. Scales, Petros Pechlivanoglou, Barbara Haas

**Affiliations:** 1 Interdepartmental Division of Critical Care, University of Toronto, Toronto, Ontario, Canada; 2 Department of Medicine, Division of Respirology, Toronto Western Hospital, University Health Network, Toronto, Ontario, Canada; 3 Sunnybrook Research Institute, Toronto, Ontario, Canada; 4 Institute of Health Policy, Management, and Evaluation, University of Toronto, Toronto, Ontario, Canada; 5 Department of Surgery, University of Toronto, Toronto, Ontario, Canada; 6 ICES, University of Toronto, Toronto, Ontario, Canada; 7 Department of Critical Care Medicine, Sunnybrook Health Sciences Centre, Toronto, Ontario, Canada; 8 Department of Medicine, University of Toronto, Toronto, Ontario, Canada; 9 Toronto Health Economic and Technology Assessment Collaborative, Toronto, Ontario, Canada; 10 Child Health Evaluative Sciences, The Hospital for Sick Children, Toronto, Ontario, Canada

## Abstract

**Importance:**

Nearly half the patients transferred from nontrauma centers to trauma centers have minor injuries, yet trauma center care is not associated with a difference in morality among patients with minor injuries. Consequently, reducing the frequency of such transfers has been postulated as a method to improve resource allocation. Currently, the economic implications of these transfers are not well understood.

**Objective:**

To estimate health care costs associated with the transfer of patients with minor injuries from nontrauma to trauma centers.

**Design, Setting, and Participants:**

This retrospective, population-based cohort study was conducted from April 1, 2009, to March 31, 2020, in Ontario, Canada. Participants included individuals aged 16 years or older who were transferred to a trauma center after presenting to a nontrauma center with a minor injury (survival >24 hours, Injury Severity Score [ISS] <16, and absence of an American College of Surgeons–defined critical injury). Statistical analysis was conducted from March 2022 to June 2024.

**Main Outcomes and Measures:**

The main outcome was total health care costs within 30 days of injury, standardized to 2015 Canadian dollars (CAD$). Propensity scoring was used to match transferred patients with controls admitted to nontrauma centers. Negative binomial models were used to estimate differences in costs between transferred patients and matched controls.

**Results:**

Of the 14 557 patients with minor injuries transferred to a trauma center (mean [SD] age, 48.1 [20.9] years; 5367 female patients [36.9%]; median ISS, 4 [IQR, 2-5]), 12 652 (86.9%) were matched with a control. Thirty days after injury, mean health care costs among transferred patients were CAD$13 540 (95% CI, CAD$13 319-CAD$13 765), a 6.5% (95% CI, 4.4%-8.5%) increase relative to controls (CAD$12 719 [95% CI, CAD$12 582-CAD$12 857]). Half the transferred patients (54.9% [7994 of 14 557]) were admitted, while the remainder were discharged after evaluation in the trauma center emergency department. Among patients admitted to a trauma center, mean 30-day costs were CAD$19 602 (95% CI, CAD$19 294-CAD$19 915), a 54.6% (95% CI, 51.5%-57.8%) increase relative to controls.

**Conclusions and Relevance:**

This cohort study of patients with minor injuries transferred from nontrauma centers to trauma centers found that the transfer of these patients was associated with increased costs to the health care system. Given the high prevalence of such transfers, these findings suggest that the development of systems to support the care of patients with minor injuries at their local hospitals is essential to the sustainability of trauma systems.

## Key Points

**Question:**

What are the health care costs associated with the transfer of patients with minor injuries from nontrauma centers to trauma centers, compared with the admission of these patients to nontrauma centers?

**Findings:**

This population-based cohort study included 14 557 patients with minor injuries transferred to trauma centers and 12 652 matched controls. The mean health care spending 30 days after injury among transferred patients was CAD$13 540 compared with CAD$12 719 among matched controls admitted to a nontrauma center, an increase of 6.5%.

**Meaning:**

This study suggests that the transfer of patients with minor injuries from nontrauma centers to trauma centers represents a substantial cost to the health care system; initiatives to reduce such transfers could result in substantial savings.

## Teaser

This cohort study estimates health care costs associated with the transfer of patients with minor injuries from nontrauma centers to trauma centers compared with the admission of these patients to nontrauma centers.

## Introduction

Trauma center care is a cost-effective intervention that is associated with reduced mortality among patients with severe injuries.^1,2,3,4,5^ However, the benefit associated with trauma center care does not extend to patients with minor injuries.^3,6^ Nonetheless, nearly half of all patients transferred from nontrauma centers to trauma centers do not have severe injuries.^7,8^

Reducing the frequency of potentially nonbeneficial transfers has been identified as a target for improved trauma system efficiency and resource allocation.^5,7,9^ Injury care is a leading contributor to health care spending, and care at a trauma center is significantly more costly than at a nontrauma center.^5,9,10,11,12,13,14,15^ However, granular evidence related to the cost of transferring patients with minor injuries from nontrauma centers to trauma centers is limited.^5,9,14,16^ As patients with minor injuries do not require intensive care or complex surgeries, it is plausible that they accrue minimal additional costs compared with patients admitted to nontrauma centers. Furthermore, care at trauma centers may be associated with benefits that lead to cost savings, including rapid assessment and discharge, decreased complications, or improved access to rehabilitation. Consequently, the true cost of such transfers is unknown. Prior to developing interventions to reduce the cost of trauma care, it is essential to understand the financial implications of transferring patients with minor injuries to trauma centers. The goal of this study was, therefore, to evaluate the cost associated with trauma center transfers among patients with minor injuries who initially presented to a nontrauma center.

## Methods

### Study Design

This was a retrospective, population-based cohort study of patients with minor injuries who were transferred from nontrauma centers to trauma centers in a large regional trauma system. This study was approved by the University of Toronto research ethics board and the ICES (formerly known as the Institute for Clinical Evaluative Sciences) Privacy and Compliance Office and reported following the Reporting of Studies Conducted Using Observational Routinely Collected Data statement.^17,18^ All costs were recorded from the perspective of the health care payer.

### Setting

This study was conducted in Ontario, Canada, between April 1, 2009, and March 31, 2020. Ontario is Canada’s most populous province (population, 14.6 million) and has 217 acute-care hospital sites, including 9 adult trauma centers.^19,20^ All medically necessary services are funded by a single-payer system with standardized reimbursement rates. Air and critical care transportation is provided by a single agency, known as Ornge.^21^

### Data Sources

Data were derived from administrative datasets held at ICES and Ornge, as well as publicly available data from municipal emergency medical services (EMS). Details of all datasets are available in eAppendix 1 and eTable 1 in Supplement 1 and have been previously described.^8,22,23^ Patients were identified using the National Ambulatory Care Reporting System and Discharge Abstract Database. ICES datasets were linked using unique encoded identifiers and analyzed at ICES.

### Study Participants

We identified all individuals 16 years of age or older who presented to the emergency department (ED) of a nontrauma center secondary to a minor injury and were subsequently transferred to a trauma center. Traumatic injuries were identified by the presence of *International Statistical Classification of Diseases and Related Health Problems, Tenth Revision* (*ICD-10*), diagnosis codes in the range S1.0 to T14.9. Patients were categorized as having a minor injury if they did not have a critical or life-threatening injury (as defined by the American College of Surgeons; eAppendix 1 and eTable 2 in Supplement 1), survived for more than 24 hours, and had an Injury Severity Score (ISS) less than 16.^7,23,24,25,26^ We excluded patients with environmental injuries and older adults (aged ≥65 years) with isolated hip fractures because these patients are not preferentially cared for within regional trauma centers.

To be conservative in our estimate of minor injuries, patients who were transferred to a trauma center and received mechanical ventilation in any ED or a blood transfusion within 24 hours of presentation were categorized as having a severe injury regardless of ISS and consequently excluded.

### Controls

To determine the costs associated with transferring patients with minor injuries to trauma centers, we matched transferred patients 1:2 with patients admitted to nontrauma centers.^27^ In this approach, we made the assumption that had patients not been transferred, they would have been admitted to a nontrauma center for evaluation and/or management. This approach provided the most conservative estimate of costs associated with trauma center care. By excluding patients discharged home from the ED of a nontrauma center as potential controls, we ensured patients with the lowest health care costs would not be included in the control group.

### Patient Characteristics

Patients were characterized by age, sex, comorbidity, frailty, receipt of chronic home-based care, nursing home residence, rural location, socioeconomic status, and whether they had a primary care clinician.^28,29^ Adjusted Clinical Groups were used to identify comorbidity level and frailty, and socioeconomic status was defined using the Ontario Marginalization Index.^30,31,32,33^ Injuries were characterized based on ISS (derived from an *ICD-10* code to Abbreviated Injury Scale algorithm), mechanism of injury, date and time, triage acuity, and ED density on arrival (details of all characteristics are available in eAppendix 1, eTable 3, and eTable 4 in Supplement 1).^26,34,35,36^

### Health Care Costs

A validated algorithm was used to capture direct health care costs using administrative health care data in Ontario.^37^ Costs captured using this method included all publicly funded costs related to hospitalizations; ED visits; same-day surgery; dialysis; inpatient mental health; physician, nonphysician, laboratory, and homecare services; prescription medications; rehabilitation; complex continuing care; nursing home care; and medical devices.

Data from municipal EMS and critical care transport services (inclusive of air transport) were used to estimate costs associated with interfacility transportation. The costs of EMS were calculated based on Toronto Paramedic Services costs.^38^ To estimate the costs of critical care transport, we calculated the median transport cost of nonseverely injured patients along each unique transfer route (consisting of a particular sending institution, receiving institution, and time frame). These costs were then applied to patients who were transferred via these routes. As critical care transports are used for selected pairs of institutions based on geography and distance, all other transfers, including all transfers within the same city, were assumed to have been performed using municipal EMS. All costs were standardized to 2015 Canadian dollars (CAD$) using the Consumer Price Index.^39^

### Outcomes

The primary outcome was total health care costs accrued within 30 days after injury. Secondary outcomes included total costs related to the initial treatment episode and sector-stratified costs. Additional outcomes included ED and hospital disposition and hospital length of stay.

### Statistical Analysis

Statistical analysis was conducted from March 2022 to June 2024. Baseline characteristics of patients who were or were not transferred to a trauma center were compared using standardized differences.^40^ Standardized differences compare the differences in mean values of baseline variables based on the SD and are not influenced by sample size.^41^ Therefore, they are less likely to identify a statistically significant difference in characteristics due to large sample size. To evaluate the costs associated with transfer to a trauma center, we conducted a propensity score–matched analysis. Multivariable logistic regression with generalized estimating equations adjusted for baseline characteristics was first used to estimate a patient’s likelihood of transfer (eAppendix 2, eTable 5, and eFigure 1 in Supplement 1). We then used a greedy matching algorithm to match each transferred patient with 2 control patients.^27^ Patients were matched based on age ±2 years, sex, home-based care use, geographic location, and propensity score (±0.2 times the SD of the logit).^42^ Finally, mean health care costs and relative cost differences between matched pairs were estimated using negative binomial models with an exchangeable correlation structure.

In addition to the primary analysis, stratified analyses were planned a priori to determine the costs of trauma center care within specific cohorts. These analyses included cohorts stratified by age, ISS, mechanism of injury, and nontrauma center type. To determine if a stratified analysis was required, we added an interaction term for each subgroup to the primary model. Because we used a greedy matching technique, if the interaction term was significant, we created a unique matched cohort to estimate the cost within each stratum. To further evaluate potential factors associated with cost, we performed a subgroup analysis based on trauma center ED disposition (admitted or discharged). Finally, to examine the robustness of our results, we performed 5 sensitivity analyses (eAppendix 2 in Supplement 1).

Statistical analysis was performed using SAS, version 9.4 (SAS Institute Inc). Standardized differences of more than 0.10 and 2-sided *P* < .05 were considered statistically significant.^40^

## Results

We identified 14 557 patients with minor injuries transferred from a nontrauma center to a trauma center (mean [SD] age, 48.1 [20.9] years; 5367 female patients [36.9%] and 9190 male patients [63.1%]; median ISS, 4 [IQR, 2-5]). Compared with patients admitted to nontrauma centers, those transferred to trauma centers were younger, more often male, less often injured in a fall, and had fewer comorbidities (Table 1^26,32^; eAppendix 3, eFigure 2, and eTables 6-8 in Supplement 1).

**Table 1.** Baseline Characteristics

Characteristic	Unmatched patients			Matched patients		
	Transferred to a trauma center (N = 14 557)	Admitted to a nontrauma center (N = 149 219)	Standardized difference	Transferred to a trauma center (n = 12 652)	Admitted to a nontrauma center (n = 22 029)	Standardized difference
**Patient characteristics**						
Age, mean (SD), y	48.1 (20.9)	66.0 (21.2)	0.85	48.9 (21.1)	50.6 (21.2)	0.08
Aged ≥65 y, No. (%)	3445 (23.7)	84 678 (56.7)	0.72	3178 (25.1)	6089 (27.6)	0.06
Sex, No. (%)						
Female	5367 (36.9)	87 486 (58.6)	0.45	4709 (37.2)	8612 (39.1)	0.04
Male	9190 (63.1)	61 733 (41.4)		7943 (62.8)	13 417 (60.9)	
Comorbidity level, No. (%)						
Low	7853 (53.9)	53 932 (36.1)	0.36	6785 (53.6)	11 414 (51.8)	0.04
Moderate	4031 (27.7)	45 284 (30.3)	0.06	3521 (27.8)	6274 (28.5)	0.01
High	2673 (18.4)	50 003 (33.5)	0.35	2346 (18.5)	4341 (19.7)	0.03
Frail, No. (%)	871 (6.0)	29 867 (20.0)	0.43	788 (6.2)	1404 (6.4)	0.01
Long-term home-based care, No. (%)	547 (3.8)	25 844 (17.3)	0.45	500 (4.0)	994 (4.5)	0.03
Nursing home, No. (%)	330 (2.3)	15 044 (10.1)	0.33	306 (2.4)	496 (2.3)	0.01
Rural, No. (%)	5018 (34.5)	21 609 (14.5)	0.48	4296 (34.0)	6968 (31.6)	0.05
Marginalization summary score, mean (SD)^a^	2.9 (0.8)	3.2 (0.8)	0.34	2.9 (0.8)	3.0 (0.7)	0.08
ISS, median (IQR)	4 (2-5)	4 (4-9)	0.20	4 (2-5)	4 (4-5)	0.06
ISS, No. (%)^b^						
<9	10 906 (74.9)	105 489 (70.7)	0.10	9483 (75.0)	16 601 (75.4)	0.01
9-15	2356 (16.2)	37 164 (24.9)	0.22	2085 (16.5)	3998 (18.1)	0.04
Unable to calculate	1295 (8.9)	6566 (4.4)	0.18	1084 (8.6)	1430 (6.5)	0.08
Mechanism of injury, No. (%)						
Gunshot wound	163 (1.1)	232 (0.2)	0.12	118 (0.9)	157 (0.7)	0.02
Cut or pierce	1281 (8.8)	3888 (2.6)	0.27	1088 (8.6)	1819 (8.3)	0.01
Fall	6126 (42.1)	116 250 (77.9)	0.79	5559 (43.9)	10 112 (45.9)	0.04
Motor vehicle collision	2559 (17.6)	7697 (5.2)	0.40	2077 (16.4)	3508 (15.9)	0.01
Pedestrian or cyclist struck	613 (4.2)	5553 (3.7)	0.03	550 (4.3)	1200 (5.4)	0.05
Other blunt	3786 (26.0)	15 085 (10.1)	0.42	3260 (25.8)	5233 (23.8)	0.05
No. of interfacility transfers (%)						
0	0	132 584 (88.9)	3.99	0	16 568 (75.2)	2.46
1	13 986 (96.1)	15 996 (10.7)	3.31	12 116 (95.8)	5284 (24.0)	2.15
≥2	571 (3.9)	639 (0.4)	0.24	536 (4.2)	177 (0.8)	0.22
Mode of first transfer, No. (%)^c^						
Municipal EMS	10 443 (71.7)	15 172 (91.2)	0.52	9076 (71.7)	4766 (87.3)	0.39
Critical care transportation	4114 (28.3)	1463 (8.8)		3576 (28.3)	695 (12.7)	
**Nontrauma center characteristics**						
Type of hospital, No. (%)						
Teaching	2576 (17.7)	20 901 (14.0)	0.10	2013 (15.9)	3593 (16.3)	0.01
Community	7343 (50.4)	118 161 (79.2)	0.63	6845 (54.1)	14 013 (63.6)	0.19
Small	4608 (31.7)	9789 (6.6)	0.67	3766 (29.8)	4371 (19.8)	0.23
CT scanner, No. (%)	9655 (66.3)	134 878 (90.4)	0.61	8685 (68.6)	17 350 (78.8)	0.23
General surgeon, No. (%)	10 901 (74.9)	138 649 (92.9)	0.51	9652 (76.3)	17 698 (80.3)	0.10
Orthopedic surgeon, No. (%)	9006 (61.9)	129 425 (86.7)	0.59	8427 (66.6)	14 607 (66.3)	0.01
ICU, No. (%)	10 739 (73.8)	137 720 (92.3)	0.51	9466 (74.8)	17 285 (78.5)	0.09

Abbreviations: CT, computed tomography; EMS, emergency medical services; ICU, intensive care unit; ISS, Injury Severity Score.

^a^ A single variable comprised of 4 distinct dimensions of marginalization; the score ranges from 1 to 5, with lower scores indicating a lower degree of marginalization and higher scores a higher degree of marginalization.^32^

^b^ A single variable representing the overall severity of an injury; the score ranges from 1 to 75, with a higher score indicating a more severe injury and a higher risk of mortality.^26^

^c^ Limited to the patients who underwent an interhospital transfer.

Most transferred patients (86.9% [n = 12 652]) were matched with at least 1 control patient admitted to a nontrauma center (Table 1). Nearly one-fourth of control patients (24.8% [5461 of 22 029]) experienced an interhospital transfer between nontrauma centers. Details of patients who could not be matched are available in eAppendix 4, eTable 9, and eTable 10 in Supplement 1.

Almost half (44.9% [5676 of 12 652]) of matched patients transferred to a trauma center were discharged from the ED of the trauma center. Compared with patients admitted to trauma centers, those discharged from the ED were more often injured by a penetrating mechanism and had injuries of lower severity (eAppendix 5, eTable 11, and eTable 12 in Supplement 1). Analysis of the subgroup of patients admitted to a trauma center demonstrated no difference in hospital mortality between patients admitted to trauma centers and matched controls (0.8% vs 0.9%; *P* = .79) (eAppendix 3 and eTable 8 in Supplement 1). However, patients admitted to trauma centers had a longer length of stay.

### Overall Costs

**Figure 1.**
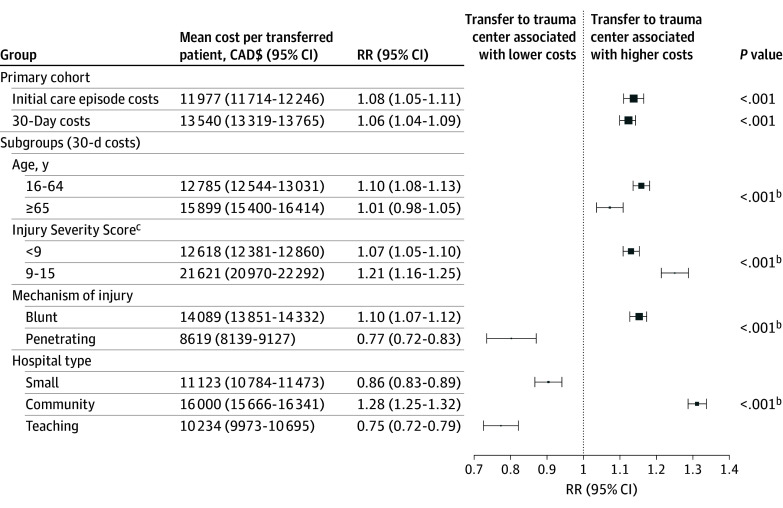
Cost Associated With Transfer of Patients With Minor Injuries to Trauma Centers, Overall and Across Subgroups^a^ Data markers are relative to population size and indicate the relative size of the subgroup. RR indicates relative risk. ^a^Standardized to 2015 Canadian dollars (CAD$). ^b^Test of significance for interaction. ^c^A single variable representing the overall severity of an injury; the score ranges from 1 to 75, with a higher score indicating a more severe injury and a higher risk of mortality.^26^

During the initial care episode, mean per-person health care costs were 8.0% higher among transferred patients compared with those treated at nontrauma centers (CAD$11 977 [95% CI, CAD$11 714-CAD$12 246] vs CAD$11 092 [95% CI, CAD$10 909-CAD$11 279]; relative risk [RR], 1.08 [95% CI, 1.05-1.11]) (Figure 1).^26^ These increased costs persisted 30 days after injury, when transfer remained associated with a 6.5% (95% CI, 4.4%-8.5%) increase in health care costs relative to matched controls (CAD$13 540 [95% CI, CAD$13 319-CAD$13 765] vs CAD$12 719 [95% CI, CAD$12 582-CAD$12 857]; RR, 1.06 [95% CI, 1.04-1.09]). Consequently, the per-person cost associated with trauma center transfer 30 days after injury was CAD$821 (95% CI, CAD$563-CAD$1085). Stratified analysis demonstrated that the cost associated with transfer varied across subgroups (Figure 1).

**Figure 2.**
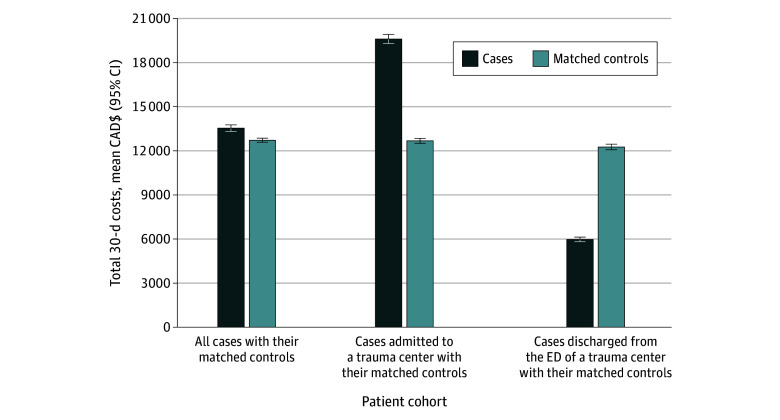
30-Day Costs for All Patients, Transferred Patients Admitted to a Trauma Center, and Transferred Patients Discharged From the Emergency Department (ED) of a Trauma Center^a^ Error bars indicate 95% CIs. ^a^Standardized to 2015 Canadian dollars (CAD$).

Given that 44.9% of transferred patients were discharged from the ED, we examined the association of ED disposition with health care spending. Among transferred patients admitted at the trauma center (54.9% [7994 of 14 557]), transfer was associated with a 54.6% (95% CI, 51.5%-57.8%) increase in 30-day costs relative to controls (CAD$19 602 [95% CI, CAD$19 294-CAD$19 915] vs CAD$12 678 [95% CI, CAD$12 509-CAD$12 849]; RR, 1.55 [95% CI, 1.52-1.58]) (Figure 2). Conversely, among transferred patients discharged from the trauma center ED, 30-day health care costs were 51.3% (95% CI, 49.8%-52.7%) lower than controls (CAD$5973 [95% CI, CAD$5819-CAD$6132] vs CAD$12 261 [95% CI, CAD$12 073-CAD$12 453]; RR, 0.49 [95% CI, 0.47-0.50]). The same association between trauma center ED disposition and cost was demonstrated across all subgroups (Table 2).

**Table 2.** Association of Transfer of Patients With Minor Injuries to Trauma Centers With Health Care Costs, Stratified by Trauma Center ED Disposition

Charactristic	Cases admitted to a trauma center			Cases discharged from the ED of a trauma center		
	Cost, CAD$ (95% CI)		RR (95% CI)	Cost, CAD$ (95% CI)		RR (95% CI)
	Cases	Matched controls		Cases	Matched controls	
Primary cohort						
Initial episode of care	18 327 (17 925-18 737)	11 050 (10 831-11 273)	1.66 (1.61-1.71)	4091 (3982-4202)	10 783 (10 531-11 042)	0.38 (0.37-0.39)
30 d costs	19 602 (19 294-19 915)	12 678 (12 509-12 849)	1.55 (1.52-1.58)	5973 (5819-6132)	12 261 (12 073-12 453)	0.49 (0.47-0.50)
Subgroups (30-d costs)						
Age category, y						
16-64	18 466 (18 130-18 807)	11 570 (11 394-11 750)	1.60 (1.56-1.63)	5797 (5625-5975)	11 326 (11 130-11 526)	0.51 (0.49-0.53)
≥65	23 007 (22 327-23 709)	15 986 (15 613-16 369)	1.44 (1.39-1.50)	6548 (6218-6896)	15 126 (14 692-15 573)	0.43 (0.41-0.46)
ISS category						
<9	18 276 (17 938-18 621)	11 846 (11 667-12 027)	1.54 (1.51-1.58)	5966 (5798-6140)	11 647 (11 450-11 847)	0.51 (0.5-0.53)
9-15	23 796 (23 126-24 486)	17 632 (17 197-18 078)	1.35 (1.30-1.40)	8708 (7735-9804)	17 164 (16 613-17 733)	0.51 (0.45-0.57)
Mechanism of injury						
Blunt	19 972 (19 649-20 300)	12 823 (12 644-13 005)	1.56 (1.53-1.59)	6078 (5911-6249)	12 375 (12 173-12 280)	0.49 (0.48-0.51)
Penetrating	14 474 (13 665-15 331)	11 296 (10 844-11 766)	1.28 (1.19-1.38)	5303 (4915-5720)	11 361 (10 886-11 857)	0.47 (0.43-0.51)
Type of hospital						
Teaching	15 050 (14 444-15 682)	13 310 (12 905-13 728)	1.13 (1.07-1.19)	4499 (4231-4783)	12 431 (12 011-12 867)	0.36 (0.34-0.39)
Community	21 596 (21 164-22 037)	12 688 (12 476-12 903)	1.70 (1.66-1.75)	6981 (6719-7252)	12 388 (12 139-12 642)	0.56 (0.54-0.59)
Small	17 642 (17 142-18 156)	12 194 (11 847-12 550)	1.45 (1.39-1.51)	5428 (5211-5654)	11 892 (11 536-12 259)	0.46 (0.43-0.48)

Abbreviations: ED, emergency department; ISS, Injury Severity Score; RR, relative risk.

### Sector-Specific Costs

**Figure 3.**
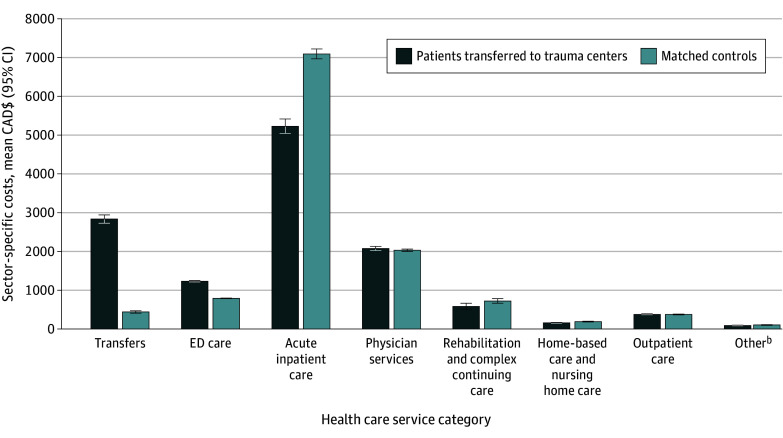
Comparison of 30-Day Sector-Specific Costs Between Patients With Minor Injuries Admitted to a Trauma Center and Their Matched Controls^a^ ED indicates emergency department. Error bars indicate 95% CIs. ^a^Standardized to 2015 Canadian dollars (CAD$). ^b^Includes publicly funded outpatient medications, medical devices, and inpatient mental health services.

A breakdown of the sector-specific health care costs demonstrated that transfer and ED costs were key factors associated with the cost of trauma center care (Figure 3; eAppendix 6 and eTable 13 in Supplement 1). Transfer to a trauma center was associated with a near 6-fold increase in transfer costs (CAD$3325 [95% CI, CAD$3237-CAD$3415] vs CAD$562 [95% CI, CAD$532-CAD$593]; RR, 5.92 [95% CI, 5.57-6.29]), and a 53.0% (95% CI, 51.2%-54.8%) increase in ED costs (CAD$1251 [95% CI, CAD$1238-CAD$1263] vs CAD$817 [95% CI, CAD$812-CAD$823]; RR, 1.53 [95% CI, 1.51-1.55]). Although acute inpatient care represented the greatest expense, on average, trauma center transfer was associated with a 28.9% (95% CI, 26.8%-30.9%) reduction in these costs (CAD$5395 [95% CI, CAD$5259-CAD$5535] vs CAD$7585 [95% CI, CAD$7486-CAD$7686]; RR, 0.71 [95% CI, 0.69-0.73]).

Examination of sector-specific costs stratified by ED disposition demonstrated that, among patients admitted to a trauma center, transfer was associated with increased costs across nearly all health care sectors, including acute inpatient care (eAppendix 6 and eTable 13 in Supplement 1). Conversely, among patients discharged from the trauma center ED, trauma center transfer was associated with a near elimination of inpatient costs during the 30 days after injury (RR, 0.09 [95% CI, 0.08-0.10]). However, even among this subgroup of patients, trauma center transfers remained associated with increased transfer and ED costs.

### Sensitivity Analyses

To examine the association of method of transportation with costs, we performed 2 sensitivity analyses assuming that either the least or most expensive mode of transportation was used for all transfers (eAppendix 7 and eTable 14 in Supplement 1). If the least expensive mode of transportation was used, transfer to a trauma center would be associated with an 8.5% (95% CI, 6.7%-10.3%) reduction in health care costs (RR, 0.92 [95% CI, 0.90-0.93]). These savings were associated with patients discharged from the ED of the trauma center. Even if the least expensive mode of transportation was used, admission of a transferred patient remained associated with a 36.5% (95% CI, 33.7%-39.3%) increase in health care costs (RR, 1.36 [95% CI, 1.34-1.39]). Results of the analysis assuming the use of the most expensive mode of transportation were consistent with the primary analysis. To examine the robustness of our model, we performed 3 analyses using different methods to account for confounding or missing data. The results were consistent with the primary analysis (eAppendix 7 and eTable 15 in Supplement 1).

## Discussion

In this study, we found that 30 days after injury, mean per-person health care costs were 6.5% higher among patients with minor injuries who were transferred from nontrauma centers to trauma centers, compared with matched controls admitted to nontrauma centers. Based on current triage practices, these data suggest that such transfers are associated with an additional CAD$1.09 million in health care spending annually. Given that nearly half the patients transferred from a nontrauma center to a trauma center have minor injuries, these findings highlight the importance of the development of interventions to facilitate the care of patients with minor injuries at local hospitals.^7,8^

The 6.5% increase in costs associated with the transfer of patients with minor injuries to trauma centers demonstrated in this study is smaller than reported previously.^9,11,14,16^ This difference is associated with differences in study design. In their study, Newgard et al^9^ compared costs across center type based on ED disposition. In their approach, the assumption was made that patients would have the same disposition regardless of center of presentation. In our study, we assumed that, had patients not been transferred to a trauma center, they would have been admitted to a nontrauma center for further evaluation. This approach biased our study toward the null; nevertheless, we found increased costs with trauma center transfers. When our analysis was stratified by ED disposition, our examination of transferred patients who were admitted to a trauma center demonstrated a similar relative increase in cost as described in the literature.^9,11,14,16^

It is likely that our results underestimate the impact of transferring patients with minor injuries to trauma centers. We demonstrated that the elevated cost associated with admitting these patients to trauma centers was partially offset by the ability of trauma centers to discharge patients at low risk from their EDs. Our findings suggest that the transfer of patients with specific minor injuries, such as those related to gunshot wounds, appeared to facilitate rapid discharge. Although our analysis was not designed to determine why specific patients were more rapidly discharged from trauma centers, trauma centers may have improved access to the advanced imaging necessary to rule out severe injuries among these patients or to clinicians who have increased familiarity with penetrating trauma. However, the transfer of these patients at low risk came at the cost of constrained resources. Each transfer required either EMS or critical care transportation services and used the resources of the trauma center. Consequently, even the transfer of patients at low risk increases strain on the trauma system, uses finite resources, and threatens access to care among severely injured patients.^7^ It is possible that interventions or additional supports at the system level (eg, telemedicine or virtual care) may provide opportunities to minimize unnecessary transfers while maintaining the benefits of trauma center expertise.^43,44,45^ Likewise, virtual consultation may allow for the identification of patients who can be safely monitored in a nontrauma center ED while awaiting imaging at a local hospital, thereby eliminating added transfer and ED costs and allowing patients at low risk to remain in their home communities.

Although prior data suggest that transferring patients with minor injuries to trauma centers was not associated with a mortality benefit, it is likely that there are patients who benefit from a lower threshold for transfer to a trauma center, such as older adults or those with preexisting frailty.^46,47^ However, our subgroup analyses indicated that younger patients had a greater association with interfacility transfer costs than older adults. This finding suggests that younger patients represent a potential target for future interventions, while acknowledging the role of trauma center care among vulnerable patients. Another argument for maintaining high transfer rates is that data demonstrate that trauma centers benefit from higher volumes.^48,49^ Therefore, while transferring patients with minor injuries may not be associated with a mortality benefit for the individual patient, it may be associated with a benefit at the population level. However, evidence suggests that significant undertriage remains.^7,8^ Consequently, it is likely that, through improvements in triage accuracy, in which transfers rates of severely injured patients are increased while those of patients with minor injuries are decreased, volumes necessary to maintain trauma center proficiency can be maintained. Finally, it is possible that the transfer of patients with minor injuries is associated with nonmortality benefits. Given the creation of care pathways focused on older adults at trauma centers, it may be that these transfers provide opportunities to optimize long-term medications and decrease the risk of future injuries.^50,51^ Likewise, given the focus of trauma centers on quality improvement, it may be that patients who receive care at these centers have a lower risk of hospital complications, such as the development of thromboembolism.^52^ To fully understand the role of trauma center care among all injured patients and determine the appropriate response to the high rates of transfers of patients with minor injuries, an evaluation of the nonmortality benefits associated with trauma center care is essential.

### Limitations

There are important limitations to consider when interpreting our results. Due to data-sharing restrictions, we could not directly link costs of critical care transport to individual patients and instead used median, maximum, and minimum costs for each transfer pathway. Yet even when we assumed the least expensive mode of transportation was used, we still demonstrated that among admitted patients, transferring patients with minor injuries to trauma centers was associated with a significant increase in health care spending. Second, it is possible that residual confounding associated with triage decisions remained. However, older patients with increased comorbidities are less likely to be transferred to trauma centers.^53^ Consequently, unmeasured confounding likely biased our study to underestimate the cost of trauma center transfers, as patients who were more likely to have longer hospital stays were less likely to be transferred. Third, while we biased our study toward overestimating injury severity, it is possible that some of the patients transferred to trauma centers had injuries that could be managed only at a trauma center. However, given the design of our study (excluding patients who received mechanical ventialtion, those who received blood transfusions, and those with specific injury patterns), it is unlikely that a small proportion of patients with injuries that necessitated trauma center care would alter the identified association. Likewise, while it is not possible to capture the indication for transfer using administrative data, trauma center transfer decisions are often based on physician heuristics.^54,55^ Consequently, factors such as mechanism of injury, hemodynamic status, and visible injury play a key role in transfer requests. We ensured that covariates reflecting these factors were included in our propensity score to minimize residual confounding associated with the indication for transfer. Fourth, we were unable to evaluate the association of interfacility transfer with individual expenses. It is likely that these transfers increase costs for patients and their families, as they are required to travel increased distances to reach a trauma center and must pay to return home. Fifth, while Ontario is the largest province in Canada and includes multiple distinct regional and geographic locations, the results may not be generalizable to all trauma networks. However, because a key aspect of regionalized care is the centralization of high-cost resources, it is likely that associations demonstrated in our study would be consistent across regional networks. Moreover, in regions that do not use a single-payer system, it is likely that cost differences would be further exaggerated, and our results may underestimate the costs associated with the transfer of patients with minor injuries in these systems.^56^

## Conclusions

This cohort study demonstrated that 30 days after injury, the transfer of patients with minor injuries from nontrauma centers to trauma centers remained associated with increased health care costs. Furthermore, the ED disposition of patients with minor injuries transferred to trauma centers significantly modified the association between transfer and healthcare costs. Admission of transferred patients to a trauma center was associated with more than a 50% increase in health care costs relative to matched controls. These findings suggest that the development of systems to minimize unnecessary transfers may result in significant health care savings.

## Notes

Supplement 1. **eAppendix 1. **Methods – Datasets and Definitions **eTable 1. **Details of Administrative Datasets **eTable 2. **Injuries Defined as Critical or Life-Threatening by the American College of Surgeons **eTable 3. **Criteria Used to Identify Environmental Injuries and Older Adults With Isolated Hip Fractures **eTable 4. **Definition of Covariates **eAppendix 2.** Methods – Analysis **eTable 5.** Multivariable Logistic Regression Estimating the Likelihood of Being Transferred to a Trauma Center **eFigure 1.** Distribution of the Logit of the Propensity Score Stratified by Transfer Status, 0=Admitted to a Non-Trauma Center, 1=Transferred to a Trauma Center **eAppendix 3. **Detailed Cohort Characteristics and Hospital Outcomes **eFigure 2. **Cohort Creation **eTable 6. **Additional Baseline Characteristics **eTable 7. **Hospital Outcomes for the Entire Cohort **eTable 8. **Hospital Outcomes Among Patients With Minor Injuries Admitted to a Trauma Center Compared to Matched Controls **eAppendix 4.** Comparison of Characteristics and Outcomes Between Matched and Unmatched Cases **eTable 9. **Differences in Baseline Characteristics Stratified by Matched Status **eTable 10. **Differences in Outcomes Stratified by Matched Status **eAppendix 5.** Comparison of Characteristics and Outcomes Between Transferred Patients Who Were and Were Not Admitted to a Trauma Center **eTable 11. **Baseline Characteristics Stratified by Admission Status **eTable 12. **Hospital Outcomes Stratified by Admission Status **eAppendix 6**. Impact of Transfer of Patients With Minor Injuries to Trauma Centers on Sector-Specific Healthcare Costs **eTable 13. **Impact of Transfer of Patients With Minor Injuries to Trauma Centers on Sector Specific-Healthcare Costs Overall and Stratified by Trauma Center ED Disposition **eAppendix 7**. Sensitivity Analyses **eTable 14.** Impact of Transfer of Patients With Minor Injuries to Trauma Centers on Healthcare Costs Assuming Either the Least or Most Expensive Form of Transport Was Used for Every Interfacility Transfer **eTable 15.** Impact of Transfer of Patients With Minor Injuries to Trauma Centers on Healthcare Costs Using Alternative Models: 1) Inverse Probability Weighted Negative Binomial Regression; 2) Use of Multiple Imputation to Estimate Missing Variables for Creation of the Propensity Score; and 3) Exclusion of All Encounters With Missing Physician Characteristics for Creation of the Propensity Score

Supplement 2.
Data Sharing Statement
