# Supplementary material for: Costs of Transfer From Nontrauma to Trauma Centers Among Patients With Minor Injuries
Source: JAMA Netw Open. 2024 Sep 20;7(9):e2434172. doi: 10.1001/jamanetworkopen.2024.34172 (PMC11415792; doi:10.1001/jamanetworkopen.2024.34172)
Supplement: Supplement 1. — eAppendix 1. Methods – Datasets and Definitions eTable 1. Details of Administrative Datasets eTable 2. Injuries Defined as Critical or Life-Threatening by the American College of Surgeons eTable 3. Criteria Used to Identify Environmental Injuries and Older Adults With Isolated Hip Fractures eTable 4. Definition of Covariates eAppendix 2. Methods – Analysis eTable 5. Multivariable Logistic Regression Estimating the Likelihood of Being Transferred to a Trauma Center eFigure 1. Distribution of the Logit of the Propensity Score Stratified by Transfer Status, 0=Admitted to a Non-Trauma Center, 1=Transferred to a Trauma Center eAppendix 3. Detailed Cohort Characteristics and Hospital Outcomes eFigure 2. Cohort Creation eTable 6. Additional Baseline Characteristics eTable 7. Hospital Outcomes for the Entire Cohort eTable 8. Hospital Outcomes Among Patients With Minor Injuries Admitted to a Trauma Center Compared to Matched Controls eAppendix 4. Comparison of Characteristics and Outcomes Between Matched and Unmatched Cases eTable 9. Differences in Baseline Characteristics Stratified by Matched Status eTable 10. Differences in Outcomes Stratified by Matched Status eAppendix 5. Comparison of Characteristics and Outcomes Between Transferred Patients Who Were and Were Not Admitted to a Trauma Center eTable 11. Baseline Characteristics Stratified by Admission Status eTable 12. Hospital Outcomes Stratified by Admission Status eAppendix 6. Impact of Transfer of Patients With Minor Injuries to Trauma Centers on Sector-Specific Healthcare Costs eTable 13. Impact of Transfer of Patients With Minor Injuries to Trauma Centers on Sector Specific-Healthcare Costs Overall and Stratified by Trauma Center ED Disposition eAppendix 7. Sensitivity Analyses eTable 14. Impact of Transfer of Patients With Minor Injuries to Trauma Centers on Healthcare Costs Assuming Either the Least or Most Expensive Form of Transport Was Used for Every Interfacility Transfer eTable 15. Impact of Transfer of Pati [file jamanetwopen-e2434172-s001.pdf]

Supplementary Online Content

Tillmann BW, Nathens AB, Guttman MP, et al. Costs of transfer from nontrauma to trauma centers among patients with minor Injuries. *JAMA Netw Open.* 2024;7(9):e2434172. doi:10.1001/jamanetworkopen.2024.34172

eAppendix 1. Methods – Datasets and Definitions
eTable 1. Details of Administrative Datasets
eTable 2. Injuries Defined as Critical or Life-Threatening by the American College of Surgeons
eTable 3. Criteria Used to Identify Environmental Injuries and Older Adults With Isolated Hip Fractures
eTable 4. Definition of Covariates
eAppendix 2. Methods – Analysis
eTable 5. Multivariable Logistic Regression Estimating the Likelihood of Being Transferred to a Trauma Center
eFigure 1. Distribution of the Logit of the Propensity Score Stratified by Transfer Status, 0=Admitted to a Non-Trauma Center, 1=Transferred to a Trauma Center
eAppendix 3. Detailed Cohort Characteristics and Hospital Outcomes
eFigure 2. Cohort Creation
eTable 6. Additional Baseline Characteristics
eTable 7. Hospital Outcomes for the Entire Cohort
eTable 8. Hospital Outcomes Among Patients With Minor Injuries Admitted to a Trauma Center Compared to Matched Controls
eAppendix 4. Comparison of Characteristics and Outcomes Between Matched and Unmatched Cases
eTable 9. Differences in Baseline Characteristics Stratified by Matched Status
eTable 10. Differences in Outcomes Stratified by Matched Status
eAppendix 5. Comparison of Characteristics and Outcomes Between Transferred Patients Who Were and Were Not Admitted to a Trauma Center
eTable 11. Baseline Characteristics Stratified by Admission Status
eTable 12. Hospital Outcomes Stratified by Admission Status
eAppendix 6. Impact of Transfer of Patients With Minor Injuries to Trauma Centers on Sector-Specific Healthcare Costs
eTable 13. Impact of Transfer of Patients With Minor Injuries to Trauma Centers on Sector Specific-Healthcare Costs Overall and Stratified by Trauma Center ED Disposition
eAppendix 7. Sensitivity Analyses
eTable 14. Impact of Transfer of Patients With Minor Injuries to Trauma Centers on Healthcare Costs Assuming Either the Least or Most Expensive Form of Transport Was Used for Every Interfacility Transfer
eTable 15. Impact of Transfer of Patients With Minor Injuries to Trauma Centers on Healthcare Costs Using Alternative Models: 1) Inverse Probability Weighted Negative Binomial Regression; 2) Use of Multiple Imputation to Estimate Missing Variables for Creation of the Propensity Score; and 3) Exclusion of All Encounters With Missing Physician Characteristics for Creation of the Propensity Score

This supplementary material has been provided by the authors to give readers additional information about their work.

© 2024 Tillmann BW et al. *JAMA Network Open.*

eAppendix 1. Methods – Datasets and Definitions

Data were obtained from several administrative databases in Ontario, Canada. All databases aside from Ornge are held at ICES

eTable 1. Details of Administrative Datasets

Database name	Description	Data derived from each database
Assistive Devices Program (ADP)	Contains information on support and funding provided to Ontario residents who have long-term physical disabilities to provide access to personalized assistive devices appropriate for their basic needs	• Costs associated with medical devices
Client Agent Program Enrollment (CAPE)	Contains information on an individual's enrollment with a specific provider or group	• Identification of patients with a primary care physician
Continuing Care Reporting System (CCRS)	Contains demographic and clinical information on patients residing in publicly funded long-term care facilities	• Identification of patients residing in nursing homes
Client Profile Database (CPRO)	Tracks applications to publicly funded long-term care facilities	• Identification of patients on nursing home waitlist
Discharge Abstract Database (DAD)	Contains information on all acute care hospitalizations in Ontario	• Identification of triage status<br>• Patient baseline characteristics<br>• Identification of patient outcomes<br>• Identification of comorbid conditions
GAPP Decision Support Systems (GAPP)	Contains information on alternative payment plans utilized for physician compensation	• Costs associated with physician services
Home Care Database (HCD)	Contains information on all publicly funded home care services in Ontario	• Identification of patients receiving homecare
Institutions database (INST)	Contains information about Ontario health care institutions funded by the ministry of Health and Long–Term Care	• Characteristics of the initial treating hospital
ICES Physician Database (IPDB)	Contains information about physician specialization and workload	• Characteristics of the initial treating hospital<br>• Characteristics of the initial treating physician
National Ambulatory Care Reporting System (NACRS)	Contains information on all emergency department visits in Ontario	• Identification of patients for study inclusion<br>• Identification of triage status<br>• Patient baseline characteristics<br>• Identification of patient outcomes

© 2024 Tillmann BW et al. *JAMA Network Open.*

National Rehabilitation Reporting System (NRS)	Contains client data collected from inpatient rehabilitation facilities and programs across Canada	• Costs associated with inpatient rehabilitation
Ontario Case Costing Initiative (OCCI)	Contains case cost data for acute inpatient, day surgery, ambulatory care, complex continuing care, and rehabilitation cases. The case costing standard ensures comparability in costing methodology across hospitals	• Costs associated with inpatient, surgical, and rehabilitation care
Ontario Drug Benefit (ODB)	Contains data related to claims for prescription medications received under the Ontario Drug Benefit program	• Costs associated with prescription medications
Ontario Health Insurance Plan Claims database (OHIP)	Captures physician services and encounters through billing claims	• Identification of patients with a primary care physician<br>• Characteristics of the initial treating hospital<br>• Characteristics of the initial treating physician<br>• Costs of physician services
Ontario Mental Health Reporting System (OMHRS)	Contains data on patients in adult inpatient mental health beds	• Costs associated with inpatient mental health services
Ontario Marginalization Index (ONMARG)	Contains information on all residents on Ontario pertaining to their social economic status	• Patient socioeconomic information
Ornge database	Contains information on all critical care prehospital and interhospital transports	• Cost associated with interfacility transfer
Registered Persons Database (RPDB)	Contains demographic information on all residents of Ontario with a health card	• Patient demographic information<br>• Identification of patient outcomes
Same Day Surgery Database (SDS)	Contains patient-level data for same day surgical procedures	• Cost associated with surgical care

© 2024 Tillmann BW et al. *JAMA Network Open.*

eTable 2. Injuries Defined as Critical or Life-Threatening by the American College of Surgeons

Injury	ICD-10-CA Code
Injury to aorta, carotid, and vertebral vessels	S15.0 – S12.9 and S25.0 – S25.9
Injury to the heart	S26.0 – S26.9
Multiple rib fractures	S22.41, S22.49, and S22.5
Injury to abdominal vasculature	S35.0 – S35.5
Open fracture with loss of distal pulse	S48.0 – S48.9, S58.0 – S58.9, S68.4, S78.0 – S78.9, S88.0 – S88.9, S98.0, T05.0 – T05.6, and T05.9
Open skull fracture	S02.001, S02.101, S02.411, S02.421, S02.431, S02.441, S02.701, S02.891, and S02.901
Head injury with a GCS <14	AIS score in the body region head ≥3
Any spinal cord injury or more than 1 vertebral column fracture	S14.0, S14.1, S14.2, S14.5, S24.0, S24.1, S24.2, S24.4, S34.0, S34.1, S34.2, S34.5, T06.0, T06.1, S12.7, S22.1, S32.7, and any combination of the following S12.0, S12.1, S12.2, S12.9, S22.0, and S32.0
Open fracture of a long bone	S42.2_1, S42.3_1, S42.4_1, S52.0_1, S52.1_1, S52.201, S52.301, S52.401, S52.701, S52.801, S52.901, T02.21, T02.41, S72.0_1, S72.1_1, S72.201, S72.301, S72.4_1, S72.701, S72.801, S72.901, S82.101, S82.201, S82.301, S82.701, S82.901, T02.31, T02.51, and T02.61,
Severe torso injury with a comorbid condition	S27.41, S27.48, S27.51, S27.58, S27.71, S27.78, S27.8, S27.9, T04.1, T04.7, T05.8, T06.5, S28, S36.02, S36.03, S36.04, S36.12, S36.13, S36.14, S36.18, S36.22, S36.23, S36.24, S36.31, S36.41, S36.42, S36.46, S36.51, S36.61, S36.71, S36.78, S36.81, S36.91, S37.02, S37.03, S37.11, S37.21, S37.61, S37.71, S37.81, S37.91, S38.2, S38.3, and S39.6<br><br>Comorbid conditions were identified using the Charlson Comorbidity Index Deyo modification
Grade IV liver laceration	S36.13, S36.14

See *Mohan D, Rosengart MR, Farris C, Cohen E, Angus DC, Barnato AE. Assessing the feasibility of the American College of Surgeons' benchmarks for the triage of trauma patients. Arch Surg. Jul 2011;146(7):786-92. doi:10.1001/archsurg.2011.43* and *Rotondo MF, Cribari C, Smith RS. Resources for Optimal Care of the Injured Patient 2014 (6th Edition). Committee on Trauma American College of Surgeons; 2014.*

© 2024 Tillmann BW et al. *JAMA Network Open.*

eTable 3. Criteria Used to Identify Environmental Injuries and Older Adults With Isolated Hip Fractures

Condition	Criteria
Burn, frost bite, foreign body through body orifice, poisonings, toxic effects, suffocation, or drowning	A patient will be considered to have one of these conditions if an ICD–10–CA diagnoses code in the range T15.0–T78.9 appears in any diagnosis field in any record during the healthcare encounter or if the injury mechanism is listed as a burn
Isolated hip fracture in an older adult	A patient will be considered to be an older adult who has have sustained an isolated hip if they meet all of the following criteria:<br>• Age ≥65<br>• Injury mechanism is listed as a fall<br>• ICD–10–CA diagnoses code of either S72.0, S72.1, or S72.2<br>• No other injuries except for superficial injuries as identified by the following ICD–10–CA codes<br>○ S*0, T09, T11.0, T13.0, T14.0

© 2024 Tillmann BW et al. *JAMA Network Open.*

eTable 4. Definition of Covariates

Variable	Definition	Sources	Type	Analysis format
**Patient and injury characteristics**				
Age	Age at time of injury	RPDB	Continuous	Continuous variable, analyzed as difference in years from the mean
Sex	Biologic sex	RPDB	Dichotomous	Male/Female
Comorbidity level	Measured using the Johns Hopkins Adjusted Clinical Groups® (ACG) System Version 10 based on health services use with a 24-month look-back window prior to the date of injury whereby the 32 aggregated diagnosis groups (ADG) are summed to create a total score (1)	DAD<br>OHIP	Categorical	Low (0 – 4)<br>Medium (5 – 9)<br>High (≥10)
Presence of frailty	Identified based on the presence of one or more diagnoses from 12 clusters of frailty-related conditions specified by the ACG system during the two years prior to their injury (2)	DAD<br>OHIP	Dichotomous	Yes/No
Chronic homecare	Defined as the receipt of publicly funded, long-term in-home supportive care within 90 days preceding the injury	HCD	Dichotomous	Yes/No
Nursing home residence	Defined as a patient who either lived in or had been accepted to a publicly funded nursing home.<br>As acceptance to a nursing home indicated a patient's functional status had declined to a point where they were no longer safe to live independently, these patients were also identified as nursing home residents	CCRS<br>CPRO	Dichotomous	Yes/No
Geographic location	Determined using postal code of residence and the "rural and small town" definition used by Statistics Canada (3)	RPDB	Dichotomous	Urban/Rural
Socioeconomic status	Determined using postal code of residence and the Canadian Marginalization Index (4)	ONMARG	Summary score is continuous, individual	Score on individual subscales<br>• 1 (Lowest)<br>• 2

© 2024 Tillmann BW et al. *JAMA Network Open.*

Variable	Definition	Sources	Type	Analysis format
			subscales are categorical	• 3<br>• 4<br>• 5 (Highest)
Primary care provider	A patient was identified as having a primary care provider if they were rostered to a primary care physician group. A previously defined algorithm was used to identify patients who were members of a primary care physician group (5)	CAPE<br>OHIP	Dichotomous	Yes/No
ISS	A validated algorithm was used to calculate the ISS for each patient based on the diagnostic codes recorded in their first hospital admission or, if not admitted, their last ED encounter (6)	DAD<br>NACRS	Categorical	1 – 8<br>9 – 15
Date and time of presentation	Date and time of presentation at the non-trauma center	NACRS	Categorical	Year<br>Day of the week<br>Time period<br>• 07:00 – 17:00<br>• 17:01 – 23:59<br>• 00:00 – 06:59
Triage acuity	Triage score assigned using the Canadian Triage Acuity Scale (7)	NACRS	Categorical	1 (Highest)<br>2<br>3<br>4<br>5 (Lowest)
ED density	The proportion of the daily total of patients who presented to the ED in the hour preceding the patient (8)	NACRS	Categorical	< 2.5%<br>2.5 – 4.99%<br>5.0 – 7.49%<br>≥ 7.5%
**Physician characteristics**				
Training	Primary specialization recorded in the ICES database. There are three training streams for ED physicians in Canada: a five-year emergency medicine residency, a two-year family medicine residency, or a three-year program that supplements a	IPDB	Categorical	Family medicine residency<br>Family medicine residency with EM fellowship<br>Five-year EM residency

© 2024 Tillmann BW et al. *JAMA Network Open.*

Variable	Definition	Sources	Type	Analysis format
	two-year family medicine residency with a one-year emergency medicine fellowship (9)			
Years in practice	Number of years after completion of training	IPDB	Categorical	< 5<br>5 – 9<br>≥ 10
Sex	Biologic sex	IPDB	Dichotomous	Male/Female
**Non-trauma center characteristics**				
Hospital type	As defined by the Ministry of Health and Long-Term Care following the guidelines set by the Joint Policy and Planning Committee (10):<br>• Small hospitals were those with less than 2,700 acute and day surgery cases during any of two of the three previous years<br>• Teaching hospitals were acute hospitals that had membership in the Council of Academic Hospitals of Ontario. These hospitals provide complex patient care, are affiliated with a medical or health sciences school, and have significant research activity<br>• Community hospitals were all other hospitals that did not meet the definition for small or teaching hospital	INST	Categorical	Small<br>Community<br>Teaching
Number of inpatient beds	Total number of inpatient beds	INST	Categorical	< 25<br>25 – 49<br>50 – 99<br>100 – 199<br>200 – 299<br>≥ 300
Number of ICU beds	Total number of ICU beds	INST	Categorical	0<br>1 – 4<br>5 – 9

© 2024 Tillmann BW et al. *JAMA Network Open.*

Variable	Definition	Sources	Type	Analysis format
				10 – 19<br>20 – 29<br>≥ 30
ED physician staffing	Defined based on which physician billing type billed for the majority of the ED procedures performed at that institution (11, 12)	OHIP	Categorical	Family medicine residency<br>Family medicine residency with EM fellowship<br>Combination of family physician types<br>Mixed (includes all combinations with Five-year EM trained phyisians)
Availability of a CT scanner	Identified as a center with at least ten billing claims for a CT scan within a year (11)	OHIP	Dichotomous	Yes/No
Presence of general surgery	Identified as a center with at least five billing claims for an appendectomy within a year (11)	OHIP	Dichotomous	Yes/No
Presence of orthopedic surgery	Identified as a center with at least five billing claims for knee arthroscopies within a year (11)	OHIP	Dichotomous	Yes/No

ISS = Injury Severity Score; ED = Emergency department; ICU = Intensive care unit; ACG = Adjusted Clinical Groups®; ADG = Aggregated diagnosis groups; RPDB = Registered Persons Database; DAD = Discharge Abstract Database; OHIP = Ontario Health Insurance Plan Claims database; HCD = Home Care Database; CCRS = Continuing Care Reporting System; CPRO = Client Profile Database; ONMARG = Ontario Marginalization Index; CAPE = Client Agent Program Enrollment; NACRS = National Ambulatory Care Reporting System; IPDB = ICES Physician Database; INST = Institutions database

1) Reid RJ, MacWilliam L, Verhulst L, Roos N, Atkinson M. Performance of the ACG case-mix system in two Canadian provinces. *Med Care*. Jan 2001;39(1):86-99.
2) Ho MM, Camacho X, Gruneir A, Bronskill SE. Overview of Cohorts, *In: Health System Use by Frail Ontario Seniors: An In-Depth Examination of Four Vulnerable Cohorts*. Institute for Clinical Evaluative Sciences; 2011.
3) du Plessis V, Beshiri R, Bollman RD, Clemenson H. Definitions of rural. In: Statistics Canada editor. *Rural and Small Town Canada Analysis Bulletin*. Ottawa, Ontario: Statistics Canada; 2001.
4) Matheson FI, Dunn JR, Smith KL, Moineddin R, Glazier RH. Development of the Canadian Marginalization Index: a new tool for the study of inequality. *Can J Public Health*. Apr 2012;103(8 Suppl 2):S12-6.
5) Glazier RH, Zagorski BM, Rayner J. Comparison of Primary Care Models in Ontario by Demographics, Care Mix and Emergency Department Use, 2008/09 to 2009/10. *ICES Investigative Report*. Toronto: Institute for Clinical Evaluative Sciences. 2012.
6) Haas B, Xiong W, Brennan-Barnes M, Gomez D, Nathens AB. Overcoming barriers to population-based injury research: development and validation of an ICD10-to-AIS algorithm. *Can J Surg*. Feb 2012;55(1):21-6. doi:10.1503/cjs.017510
7) J Murray M. The Canadian Triage and Acuity Scale: A Canadian perspective on emergency department triage. *Emerg Med (Fremantle)*. Feb 2003;15(1):6-10.

© 2024 Tillmann BW et al. *JAMA Network Open.*

8) Forster AJ, Stiell I, Wells G, Lee AJ, van Walraven C. The effect of hospital occupancy on emergency department length of stay and patient disposition. *Acad Emerg Med*. Feb 2003;10(2):127-33
9) Bhimani M, Dickie G, McLeod S, Kim D. Emergency medicine training demographics of physicians working in rural and regional southwestern Ontario emergency departments. *CJEM*. Nov 2007;9(6):449-52.
10) Canadian Institute for Health Information. Hospital Report: Emergency Department Care 2007. CIHI. Accessed January 31, 2023. https://secure.cihi.ca/free_products/OHA_ED_07_EN_final_secure.pdf
11) Gomez D, Haas B, de Mestral C, et al. Institutional and provider factors impeding access to trauma center care: an analysis of transfer practices in a regional trauma system. *The journal of trauma and acute care surgery*. 2012;73(5):1288-1293. doi:10.1097/TA.0b013e318265cec2
12) Tillmann BW, Nathens AB, Guttman MP, et al. Hospital resources do not predict accuracy of secondary trauma triage: A population-based analysis. *J Trauma Acute Care Surg*. Feb 2020;88(2):230-241. doi:10.1097/TA.0000000000002552

© 2024 Tillmann BW et al. *JAMA Network Open.*

eAppendix 2. Methods – Analysis

Details of the propensity match

Multivariable logistic regression with generalized estimating equations was used to estimate a patient's likelihood of transfer and assign each patient a propensity score representing their likelihood of transfer to a trauma center. The model was adjusted for patient (age, sex, comorbidity, frailty, homecare, nursing home residence, geographic location, socioeconomic status) and injury (ISS, mechanism, triage acuity, date and time, ED density) characteristics known to impact the probability of transfer.[1] In addition to these variables, characteristics of the treating physician and available hospitals resources likely impact a patient's probability of transfer.[1, 2] Therefore physician (sex, training, years in practice) and hospital (type, number of hospital and intensive care unit beds, ED physician staffing, availability of a CT scanner, surgical support) characteristics were also included in the model.[3-9]

Data related to the treating physician was missing in 19.8% (n=32,469) of encounters, for socioeconomic status in 1.4% (n=829) of encounters, and for non-trauma hospital type in 0.9% (n=1,526) of encounters, and the ISS could not be calculated in 4.8% (n=7,861). As encounters in which physician data is missing are at higher risk for transfer, we created a missing category for all physician characteristics.[1, 10] Likewise, as ISS may not be missing at random, an "unknown" category was created. Episodes missing data related to non-trauma hospital type were excluded as they had minimal information regarding all hospital characteristics. A complete-case analysis was used to create the propensity score.

1) Tillmann BW, Nathens AB, Guttman MP, et al. The impact of referring hospital resources on interfacility overtriage: A population-based analysis. *Injury*. Jan 17 2024;55(3):111332. doi:10.1016/j.injury.2024.111332
2) Mohan D, Barnato AE, Rosengart MR, et al. Trauma triage in the emergency departments of nontrauma centers: an analysis of individual physician caseload on triage patterns. *J Trauma Acute Care Surg*. Jun 2013;74(6):1541-7. doi:10.1097/TA.0b013e31828c3f75
3) Bhimani M, Dickie G, McLeod S, Kim D. Emergency medicine training demographics of physicians working in rural and regional southwestern Ontario emergency departments. *CJEM*. Nov 2007;9(6):449-52.
4) Lurie N, Slater J, McGovern P, Ekstrum J, Quam L, Margolis K. Preventive care for women. Does the sex of the physician matter? *N Engl J Med*. Aug 1993;329(7):478-82. doi:10.1056/NEJM199308123290707
5) Bertakis KD, Helms LJ, Callahan EJ, Azari R, Robbins JA. The influence of gender on physician practice style. *Med Care*. Apr 1995;33(4):407-16. doi:10.1097/00005650-199504000-00007
6) Berthold HK, Gouni-Berthold I, Bestehorn KP, Böhm M, Krone W. Physician gender is associated with the quality of type 2 diabetes care. *J Intern Med*. Oct 2008;264(4):340-50. doi:10.1111/j.1365-2796.2008.01967.x
7) Mohan D, Rosengart MR, Farris C, Fischhoff B, Angus DC, Barnato AE. Sources of non-compliance with clinical practice guidelines in trauma triage: a decision science study. *Implement Sci*. Oct 2012;7:103. doi:10.1186/1748-5908-7-103
8) Gomez D, Haas B, de Mestral C, et al. Institutional and provider factors impeding access to trauma center care: an analysis of transfer practices in a regional trauma system. *The journal of trauma and acute care surgery*. 2012;73(5):1288-1293. doi:10.1097/TA.0b013e318265cec2
9) Tillmann BW, Nathens AB, Guttman MP, et al. Hospital resources do not predict accuracy of secondary trauma triage: A population-based analysis. *J Trauma Acute Care Surg*. Feb 2020;88(2):230-241. doi:10.1097/TA.0000000000002552
10) Haukoos JS, Newgard CD. Advanced statistics: missing data in clinical research--part 1: an introduction and conceptual framework. *Acad Emerg Med*. Jul 2007;14(7):662-8. doi:10.1197/j.aem.2006.11.037

© 2024 Tillmann BW et al. *JAMA Network Open.*

eTable 5. Multivariable Logistic Regression Estimating the Likelihood of Being Transferred to a Trauma Center

	Beta-coefficient (95% CI)
Intercept	-1.38 (-1.79 – -0.97)
Patient characteristics	
Age, one year change from the mean	-0.02 (-0.02 – -0.02)
Male sex	0.20 (0.15 – 0.25)
Comorbidity level	
Low	–
Moderate	-0.03 (-0.07 – 0.00)
High	-0.08 (-0.14 – -0.03)
Frail	-0.22 (-0.32 – -0.12)
Chronic Homecare	-0.55 (-0.68 – -0.42)
Nursing home resident	-0.19 (-0.3 – -0.09)
Instability quintile	
1 (lowest)	0.03 (-0.05 – 0.10)
2	-0.02 (-0.07 – 0.04)
3	–
4	-0.01 (-0.07 – 0.04)
5 (highest)	-0.02 (-0.10 – 0.06)
Deprivation quintile	
1 (lowest)	0.01 (-0.05 – 0.08)
2	-0.02 (-0.08 – 0.03)
3	–
4	-0.03 (-0.08 – 0.01)
5 (highest)	-0.03 (-0.08 – 0.03)
Dependency quintile	
1 (lowest)	-0.06 (-0.12 – 0.01)
2	0.01 (-0.05 – 0.06)
3	–
4	0.02 (-0.03 – 0.07)
5 (highest)	-0.05 (-0.11 – 0.00)
Ethnic concentration	
1 (lowest)	0.01 (-0.05 – 0.07)
2	0.01 (-0.04 – 0.06)
3	–
4	-0.02 (-0.08 – 0.04)
5 (highest)	-0.03 (-0.10 – 0.05)
Rural	-0.12 (-0.19 – -0.05
ISS	
<9	–
9–15	-0.15 (-0.26 – -0.04)
Unable to calculate	0.50 (0.29 – 0.71)

© 2024 Tillmann BW et al. *JAMA Network Open.*

	Beta-coefficient (95% CI)
Mechanism of injury	
MVC	–
GSW	0.79 (0.44 – 1.15)
Cut/pierce	-0.13 (-0.28 – 0.02)
Fall	-1.01 (-1.13 – -0.90)
Pedestrian/cyclist struck	-0.60 (-0.71 – -0.49)
Other blunt mechanism	-0.47 (-0.58 – -0.37)
Triage acuity score	
1 (highest acuity)	0.81 (0.66 – 0.97)
2	0.32 (0.24 – 0.40)
3	–
4	0.09 (0.02 – 0.17)
5 (lowest acuity)	0.19 (-0.05 – 0.43)
Missing	-0.06 (-0.40 – 0.29)
ED density on arrival	
<2.5	-0.04 (-0.09 – 0.01)
2.5 – 4.9	–
5.0 – 7.4	0.01 (-0.03 – 0.05)
≥7.5	0.00 (-0.05 – 0.06)
Time of hospital presentation	
07:00 – 16:59	–
17:00 – 23:59	-0.08 (-0.14 – -0.03)
00:00 – 06:59	0.08 (0.01 – 0.15)
Day of the week	
Monday	–
Tuesday	-0.08 (-0.14 – -0.01)
Wednesday	-0.11 (-0.18 – -0.03)
Thursday	-0.10 (-0.17 – -0.03)
Friday	-0.10 (-0.16 – -0.04)
Saturday	-0.08 (-0.14 – -0.02)
Sunday	0.04 (-0.01 – 0.09)
Year	
2009	–
2010	0.12 (0.03 – 0.20)
2011	0.26 (0.08 – 0.43)
2012	0.28 (0.13 – 0.43)
2013	0.32 (0.17 – 0.46)
2014	0.29 (0.15 – 0.42)
2015	0.35 (0.21 – 0.48)
2016	0.37 (0.24 – 0.51)
2017	0.31 (0.13 – 0.49)
2018	0.18 (-0.01 – 0.38)
2019	0.20 (-0.01 – 0.40)
2020	0.12 (0.03 – 0.20)

© 2024 Tillmann BW et al. *JAMA Network Open.*

	Beta-coefficient (95% CI)
**Physician characteristics**	
Sex	
Male	–
Female	0.09 (0.03 – 0.15)
Missing	0.68 (0.32 – 1.04)
Specialty	
Family medicine	0.09 (0.03 – 0.15)
Family medicine with EM fellowship	–
Five–year EM specialist	-0.03 (-0.20 – 0.13)
Missing	-0.97 (-1.13 – -0.81)
Years in practice	
<5	0.13 (0.07 – 0.19)
5 – 9	0.00 (-0.05 – 0.05)
≥10	–
Unknown	0.83 (0.48 – 1.19)
**Hospital Characteristics**	
Type of hospital	
Teaching	1.06 (0.15 – 1.96)
Community	–
Small	0.42 (-0.05 – 0.90)
Unspecified	-0.36 (-1.29 – 0.57)
Number of Hospital beds	
<25	0.41 (-0.34 – 1.15)
25 – 49	0.73 (0.11 – 1.36)
50 – 99	0.57 (0.08 – 1.07)
100 – 199	0.15 (-0.23 – 0.53)
200 – 299	–
≥300	0.23 (-0.22 – 0.68)
No CT scanner	-0.36 (-0.82 – 0.10)
No General Surgery	0.47 (-0.24 – 1.18)
No Orthopedic Surgery	0.32 (-0.10 – 0.73)
Number of ICU beds	
0	0.42 (-0.40 – 1.25)
1 – 4	0.77 (0.16 – 1.38)
5 – 9	0.02 (-0.26 – 0.30)
10 – 19	–
20 – 29	-0.03 (-0.30 – 0.25)
≥30	-0.88 (-1.67 – -0.09)
ED staffing mix	
Family MD	0.04 (-0.12 – 0.21)
Family MD with extra year of EM training	-0.09 (-0.24 – 0.06)
Combination Family MD types	–
Mixed	0.10 (-0.09 – 0.28)
Unknown	-0.16 (-0.48 – 0.15)

© 2024 Tillmann BW et al. *JAMA Network Open.*

eFigure 1. Distribution of the Logit of the Propensity Score Stratified by Transfer Status, 0=Admitted to a Non-Trauma Center, 1=Transferred to a Trauma Center

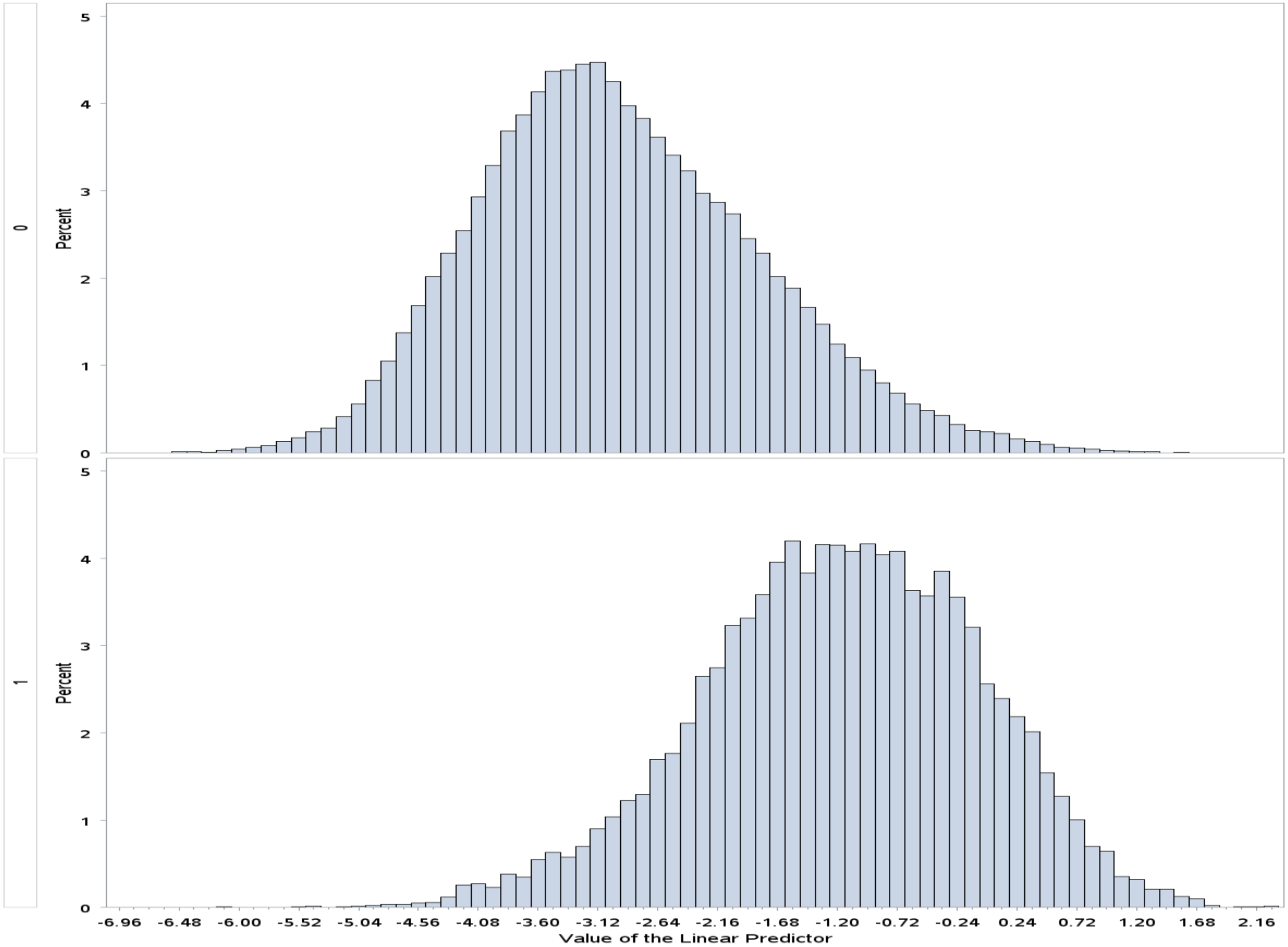

© 2024 Tillmann BW et al. *JAMA Network Open.*

Sensitivity analyses

We performed five sensitivity analyses to examine the robustness of our results. In the first two analyses, transfer costs were replaced with the lowest and highest possible costs. In most cases this meant critical care transport costs were replaced with EMS costs or vice versa. In the final three sensitivity analysis we used alternative models to ensure our results were robust to the exclusion of unmatched patients and missing data. Specifically, we a) used an inverse probability weighted negative binomial model; b) used multiple imputation to estimate values for all missing covariates; and c) excluded physician characteristics from the model.

© 2024 Tillmann BW et al. *JAMA Network Open.*

eAppendix 3. Detailed Cohort Characteristics and Hospital Outcomes

eFigure 2. Cohort Creation

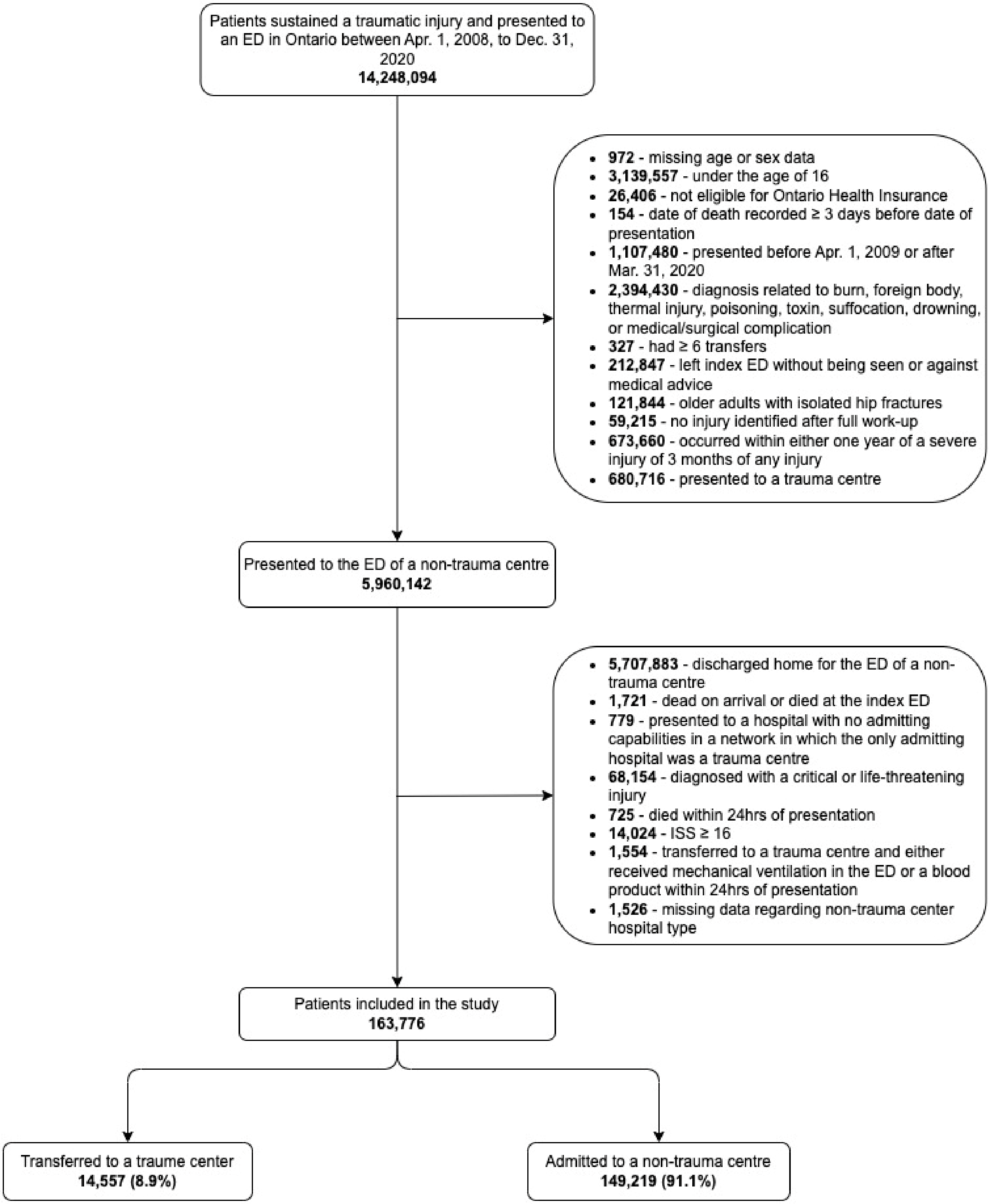

© 2024 Tillmann BW et al. *JAMA Network Open.*

eTable 6. Additional Baseline Characteristics

	Unmatched				Matched		
	All patients (n=163,776)	Transferred to a trauma center (n=14,557)	Admitted to a non-trauma center (n=149,219)	Stand. Diff.	Transferred to a trauma center (n=12,652)	Admitted to a non-trauma center (n=22,029)	Stand. Diff.
Patient Characteristics							
Regular primary care provider, n (%)	155,333 (94.8)	13,332 (91.6)	142,001 (95.2)	0.14	11,670 (92.2)	20,349 (92.4)	0.01
Triage score, n (%)							
1 (highest acuity)	3,943 (2.4)	804 (5.5)	3,139 (2.1)	0.18	662 (5.2)	1,334 (6.1)	0.04
2	53,108 (32.4)	6,009 (41.3)	47,099 (31.6)	0.20	5,270 (41.7)	9,517 (43.2)	0.03
3	89,610 (54.7)	6,034 (41.5)	83,576 (56.0)	0.29	5,361 (42.4)	8,914 (40.5)	0.04
4	16,110 (9.8)	1,566 (10.8)	14,544 (9.7)	0.03	1,257 (9.9)	2,059 (9.3)	0.02
5 (lowest acuity)	640 (0.4)	67 (0.5)	573 (0.4)	0.01	60 (0.5)	121 (0.5)	0.01
ED density, mean (±SD)	4.9 (2.6)	5.0 (3.3)	4.9 (2.6)	0.04	4.9 (3.2)	4.9 (3.2)	<0.01
Time of presentation, n (%)							
Weekday	31,813 (39.5)	5,395 (37.1)	58,895 (39.5)	0.05	4,638 (36.7)	8,023 (36.4)	<0.01
Evening or weekend	80,334 (49.1)	7,279 (50.0)	73,055 (49.0)	0.02	6,368 (50.3)	11,208 (50.9)	0.01
Night	19,152 (11.7)	1,883 (12.9)	17,269 (11.6)	0.04	1,646 (13.0)	2,798 (12.7)	0.01
Instability quintile, n (%)							
1 (lowest)	22,669 (13.8)	2,254 (15.5)	20,415 (13.7)	0.05	2,000 (15.8)	3,329 (15.1)	0.02
2	27,305 (16.7)	3,001 (20.6)	24,304 (16.3)	0.11	2,696 (21.3)	4,278 (19.4)	0.05
3	30,344 (18.5)	3,145 (21.6)	27,199 (18.2)	0.08	2,829 (22.4)	4,952 (22.5)	<0.01
4	35,342 (21.6)	3,080 (21.2)	32,262 (21.6)	0.01	2,816 (22.3)	4,941 (22.4)	<0.01
5 (highest)	4,5864 (28.0)	2,610 (17.9)	43,254 (29.0)	0.26	2,311 (18.3)	4,529 (20.6)	0.06
Deprivation quintile, n (%)							
1 (lowest)	28,544 (17.4)	2,599 (17.9)	25,945 (17.4)	0.01	2,281 (18.0)	3,813 (17.3)	0.02
2	30,599 (18.7)	2,883 (19.8)	27,716 (18.6)	0.03	2,611 (20.6)	4,389 (19.9)	0.02
3	31,769 (19.4)	3,022 (20.8)	28,747 (19.3)	0.04	2,725 (21.5)	4,763 (21.6)	<0.01
4	33,246 (20.3)	2,750 (18.9)	30,496 (20.4)	0.04	2,503 (19.8)	4,520 (20.5)	0.02
5 (highest)	37,366 (22.8)	2,836 (19.5)	34,530 (23.1)	0.09	2,532 (20.0)	4,544 (20.6)	0.02
Dependency quintile, n (%)							
1 (lowest)	24,907 (15.2)	1,944 (13.4)	22,963 (15.4)	0.06	1,739 (13.7)	3,322 (15.1)	0.04
2	26,600 (16.2)	2,537 (17.4)	24,063 (16.1)	0.03	2,237 (17.7)	3,851 (17.5)	0.01
3	27,772 (17.0)	2,826 (19.4)	24,946 (16.7)	0.07	2,486 (19.6)	4,063 (18.4)	0.03

© 2024 Tillmann BW et al. *JAMA Network Open.*

	Unmatched				Matched		
	All patients (n=163,776)	Transferred to a trauma center (n=14,557)	Admitted to a non-trauma center (n=149,219)	Stand. Diff.	Transferred to a trauma center (n=12,652)	Admitted to a non-trauma center (n=22,029)	Stand. Diff.
4	31,362 (19.2)	2,974 (20.4)	28,388 (19.0)	0.04	2,656 (21.0)	4,792 (21.8)	0.02
5 (highest)	50,883 (31.1)	3,809 (26.2)	47,074 (31.5)	0.12	3,534 (27.9)	6,001 (27.2)	0.02
Ethnic concentration, n (%)							
1 (lowest)	38,743 (23.7)	5,182 (35.6)	33,561 (22.5)	0.29	4,753 (37.6)	7,644 (34.7)	0.06
2	35,179 (21.5)	3,679 (25.3)	31,500 (21.1)	0.10	3,276 (25.9)	5,332 (24.2)	0.04
3	30,029 (18.3)	2,195 (15.1)	27,834 (18.7)	0.10	1,904 (15.0)	3,437 (15.6)	0.02
4	28,306 (17.3)	1,627 (11.2)	26,679 (17.9)	0.19	1,451 (11.5)	2,889 (13.1)	0.05
5 (highest)	29,267 (17.9)	1,407 (9.7)	27,860 (18.7)	0.26	1,268 (10.0)	2,727 (12.4)	0.07
Transferring Physician Characteristics							
Sex, n (%)							
Female	32,196 (19.7)	3,207 (22.0)	28,989 (19.4)	0.06	2,837 (22.4)	4,117 (18.7)	0.09
Male	99,264 (60.6)	6,831 (46.9)	92,433 (61.9)	0.31	6,063 (47.9)	10,900 (49.5)	0.03
Missing	32,316 (19.7)	4,519 (31.0)	27,797 (18.6)	0.29	3,752 (29.7)	7,012 (31.8)	0.05
Training, n (%)							
Family medicine residency	47,195 (28.8)	5,589 (38.4)	41,606 (27.9)	0.22	4,862 (38.4)	8,074 (36.7)	0.04
Family medicine residency with EM fellowship	62,555 (38.2)	3,372 (23.2)	59,183 (39.7)	0.36	3,106 (24.5)	5,562 (25.2)	0.02
Five–year EM residency	12,899 (7.9)	913 (6.3)	11,986 (8.0)	0.07	783 (6.2)	1,051 (4.8)	0.06
Missing	41,127 (25.1)	4,683 (32.2)	36,444 (24.4)	0.17	3,901 (30.8)	7,342 (33.3)	0.05
Years in practice, n (%)							
<5	30,052 (18.4)	2,480 (17.0)	27,572 (18.5)	0.04	2,185 (17.3)	3,851 (17.5)	0.01
5 – 9	26,682 (16.3)	1,755 (12.1)	24,927 (16.7)	0.13	1,584 (12.5)	2,629 (11.9)	0.02
≥10	74,573 (45.5)	5,774 (39.7)	68,799 (46.1)	0.13	5,107 (40.4)	8,502 (38.6)	0.04
Missing	32,469 (19.8)	4,548 (31.2)	27,921 (18.7)	0.29	3,776 (29.8)	7,047 (32.0)	0.05
Non–Trauma Center Characteristics							
Hospital beds, n (%)							
<25	12,569 (7.7)	3,481 (23.9)	9,088 (6.1)	0.52	2,792 (22.1)	3,818 (17.3)	0.12
25 – 49	15,892 (9.7)	3,994 (27.4)	11,898 (8.0)	0.53	1,821 (27.0)	2,549 (21.8)	0.12
50 – 99	19,821 (12.1)	1,909 (13.1)	17,912 (12.0)	0.03	1,694 (13.4)	3,500 (15.9)	0.07
100 – 199	36,431 (22.2)	1,402 (9.6)	35,029 (23.5)	0.38	1,364 (10.8)	3,072 (13.9)	0.10
200 – 299	39,004 (23.8)	1,581 (10.9)	37,423 (25.1)	0.38	1,546 (12.2)	3,157 (14.3)	0.06

© 2024 Tillmann BW et al. *JAMA Network Open.*

	Unmatched				Matched		
	All patients (n=163,776)	Transferred to a trauma center (n=14,557)	Admitted to a non-trauma center (n=149,219)	Stand. Diff.	Transferred to a trauma center (n=12,652)	Admitted to a non-trauma center (n=22,029)	Stand. Diff.
≥300	35,470 (21.7)	2,013 (13.8)	33,457 (22.4)	0.22	1,953 (15.4)	3,605 (16.4)	0.03
ICU beds, n (%) [a]							
1 – 4	12,002 (8.1)	3,556 (33.1)	8,446 (6.1)	0.72	3,048 (32.2)	3,905 (22.6)	0.22
5 – 9	28,041 (18.9)	1,806 (16.8)	26,235 (19.0)	0.06	1,750 (18.5)	4,177 (24.2)	0.14
10 – 19	45,803 (30.9)	2,120 (19.7)	43,683 (31.7)	0.28	2,068 (21.8)	3,944 (22.8)	0.02
20 – 29	34,198 (23.0)	1,681 (15.7)	32,517 (23.6)	0.20	1,633 (17.3)	3,427 (19.8)	0.07
≥30	22,971 (15.5)	994 (9.3)	21,977 (16.0)	0.20	967 (10.2)	1,832 (10.6)	0.01
ED staffing mix, n (%)							
Family physicians	15,149 (9.3)	2,686 (18.5)	12,463 (8.4)	0.30	2,213 (17.5)	4,203 (19.1)	0.04
Family physicians with extra EM training	15,107 (9.2)	524 (3.6)	14,583 (9.8)	0.25	497 (3.9)	1,382 (6.3)	0.11
Combination of family physician types	43,005 (26.3)	2,212 (15.2)	40,793 (27.3)	0.30	2,039 (16.1)	4,512 (20.5)	0.11
Mixed	89,009 (54.4)	8,935 (61.4)	80,074 (53.7)	0.16	7,831 (61.9)	11,709 (53.2)	0.18
Unknown	1,506 (0.9)	200 (1.4)	1,306 (0.9)	0.05	72 (0.6)	223 (1.0)	0.05

[a] Limited to the patients who presented to an NTC with an ICU

SD = Standardized differences; ISS = Injury severity score; GSW = Gunshot wound; MVC = Motor vehicle collision; EM = emergency medicine; ED = Emergency department; ICU = Intensive care unit

© 2024 Tillmann BW et al. *JAMA Network Open.*

eTable 7. Hospital Outcomes for the Entire Cohort

	All patients (n=163,776)	Transferred to a trauma center (n=14,557)	Admitted to a non-trauma center (n=149,219)	p-value
ED disposition, n (%)				
Admitted	157,213 (96.0)	7,994 (54.9)	149219 (0)	<0.001 [a]
Discharged to home	6,563 (4.1)	6563 (45.1)	0	
Hospital disposition, n (%)				
Died	3,628 (2.2)	62 (0.4)	3,566 (2.4)	<0.001 [a]
Home without additional supports	73,865 (45.1)	11,073 (76.1)	62,792 (42.1)	
Home with additional supports	40,233 (24.6)	1,778 (12.2)	38,455 (25.8)	
Inpatient rehabilitation	29,252 (17.9)	594 (4.1)	28,658 (19.2)	
Nursing home	13,967 (8.5)	463 (3.2)	13,504 (9.1)	
Other	2,577 (1.6)	333 (2.3)	2,244 (1.5)	
Length of stay, median (IQR)	5 (2 – 10)	1 (1 – 5)	5 (2 – 11)	<0.001 [b]

[a] Compared using the Chi-square test
[b] Compared using the Wilcoxon-Mann Whitney test

ED = Emergency department

© 2024 Tillmann BW et al. *JAMA Network Open.*

eTable 8. Hospital Outcomes Among Patients With Minor Injuries Admitted to a Trauma Center Compared to Matched Controls

	Admitted to a trauma center (n=7,232)	Admitted to a non-trauma center (n=14,090)	p-value
Hospital disposition, n (%)			
Died	59 (0.8)	123 (0.9)	<0.001[a]
Home without additional supports	4,597 (63.6)	9,230 (65.5)	
Home with additional supports	1,576 (21.8)	2,791 (19.8)	
Inpatient rehabilitation	553 (7.7)	1,024 (7.3)	
Nursing home	254 (3.5)	474 (3.4)	
Other	193 (2.7)	448 (3.2)	
Length of stay, median (IQR)	4 (2 – 9)	3 (2 – 6)	<0.001[b]

[a] Compared using the marginal homogeneity test
[b] Compared using the Wilcoxon signed rank sum test

© 2024 Tillmann BW et al. *JAMA Network Open.*

eAppendix 4. Comparison of Characteristics and Outcomes Between Matched and Unmatched Cases

eTable 9. Differences in Baseline Characteristics Stratified by Matched Status

	Matched (n=12,652)	Unmatched (n=1,905)	SD
Patient Characteristics			
Age, mean (±SD)	48.9 (21.1)	42.9 (19.1)	0.30
Age ≥ 65, n (%)	3,178 (25.1)	267 (14.0)	0.28
Female, n (%)	4,709 (37.2)	658 (34.5)	0.06
Comorbidity level, n (%)			
Low	6,785 (53.6)	1,068 (56.1)	0.05
Moderate	3,521 (27.8)	510 (26.8)	0.02
High	2,346 (18.5)	327 (17.2)	0.04
Frail, n (%)	788 (6.2)	83 (4.4)	0.08
Chronic homecare, n (%)	500 (4.0)	47 (2.5)	0.08
Nursing home, n (%)	306 (2.4)	24 (1.3)	0.09
Rural, n (%)	4,296 (34.0)	722 (37.9)	0.08
Marginalization score, mean (±SD)	2.9 (0.8)	2.9 (0.8)	0.05
Regular primary care provider, n (%)	11,670 (92.2)	1,662 (87.2)	0.17
ISS, median (IQR)	4 (2 – 5)	4 (1 – 4)	0.11
ISS, n (%)			
<9	9,483 (75.0)	1,423 (74.7)	0.01
9 – 15	2,085 (16.5)	271 (14.2)	0.06
Unable to calculate	1,084 (8.6)	211 (11.1)	0.08
Mechanism of injury, n (%)			
GSW	118 (0.9)	45 (2.4)	0.11
Cut/pierce	1,088 (8.6)	193 (10.1)	0.05
Fall	5,559 (43.9)	567 (29.8)	0.30
MVC	2,077 (16.4)	482 (25.3)	0.22
Pedestrian/cyclist struck	550 (4.3)	63 (3.3)	0.05
Other blunt mechanism	3,260 (25.8)	526 (27.6)	0.04
Triage score, n (%)			
1 (highest acuity)	662 (5.2)	142 (7.5)	0.09
2	5,270 (41.7)	739 (38.8)	0.06
3	5,361 (42.4)	673 (35.3)	0.14
4	1,257 (9.9)	309 (16.2)	0.19
5 (lowest acuity)	60 (0.5)	7 (0.4)	0.02
ED arrival density, mean (±SD)	4.9 (3.2)	5.6 (3.9)	0.17
Time of hospital presentation, n (%)			
Weekday	4,638 (36.7)	757 (39.7)	0.06
Evening or weekend	6,368 (50.3)	911 (47.8)	0.05
Night	1,646 (13.0)	237 (12.4)	0.02
No. of interhospital transfers, n (%)			
1	12,116 (95.8)	1,870 (98.2)	0.14
≥2	536 (4.2)	35 (1.8)	
Mode of first interhospital transfer, n (%)			
Municipal EMS	9,076 (71.7)	1,367 (71.8)	<0.01
Critical care transport	3,576 (28.3)	538 (28.2)	

© 2024 Tillmann BW et al. *JAMA Network Open.*

	Matched (n=12,652)	Unmatched (n=1,905)	SD
**Transferring Physician Characteristics**			
Sex, n (%)			
Female	2,837 (22.4)	370 (19.4)	0.07
Male	6,063 (47.9)	768 (40.3)	0.15
Missing	3,752 (29.7)	767 (40.3)	0.22
Training, n (%)			
Family medicine residency	4,862 (38.4)	727 (38.2)	0.01
Family medicine residency with EM fellowship	3,106 (24.5)	266 (14.0)	0.27
Five–year EM residency	783 (6.2)	130 (6.8)	0.03
Missing	3,901 (30.8)	782 (41.0)	0.21
Years in practice, n (%)			
<5	2,185 (17.3)	295 (15.5)	0.05
5 – 9	1,584 (12.5)	171 (9.0)	0.11
≥10	5,107 (40.4)	667 (35.0)	0.11
Missing	3,776 (29.8)	772 (40.5)	0.23
**Non–Trauma Center Characteristics**			
Type of hospital, n (%)			
Teaching	2,013 (15.9)	563 (29.6)	0.33
Community	6,845 (54.1)	498 (26.1)	0.60
Small	3,766 (29.8)	842 (44.2)	0.30
Number of hospital beds, n (%)			
<25	2,792 (22.1)	689 (36.2)	0.31
25 – 49	1,821 (27.0)	691 (36.3)	0.22
50 – 99	1,694 (13.4)	215 (11.3)	0.06
100 – 199	1,364 (10.8)	38 (2.0)	0.37
200 – 299	1,546 (12.2)	35 (1.8)	0.41
≥300	1,953 (15.4)	60 (3.1)	0.43
CT scanner, n (%)	8,685 (68.6)	970 (50.9)	0.37
General surgeon, n (%)	9,652 (76.3)	1,249 (65.6)	0.24
Orthopedic surgeon, n (%)	8,427 (66.6)	579 (30.4)	0.78
ICU, n (%)	9,466 (74.8)	1,273 (66.8)	0.18
ICU beds, n (%) [a]			
1 – 4	3,048 (32.2)	508 (39.9)	0.16
5 – 9	1,750 (18.5)	56 (4.4)	0.45
10 – 19	2,068 (21.8)	52 (4.1)	0.55
20 – 29	1,633 (17.3)	48 (3.8)	0.45
≥30	967 (10.2)	27 (2.1)	0.34
ED staffing mix, n (%)			
Family physicians	2,213 (17.5)	473 (24.8)	0.18
Family physicians with extra EM training	497 (3.9)	27 (1.4)	0.16
Combination of family physician types	2,039 (16.1)	173 (9.1)	0.21
Mixed	7,831 (61.9)	1,104 (58.0)	0.08
Unknown	72 (0.6)	128 (6.7)	0.33

[a] Limited to the patients who presented to a non-trauma center with an ICU

© 2024 Tillmann BW et al. *JAMA Network Open.*

eTable 10. Differences in Outcomes Stratified by Matched Status

	Matched (n=12,652)	Unmatched (n=1,905)	p-value
ED disposition, n (%)			
Admitted	6,979 (55.1)	1,018 (53.4)	0.16 [a]
Discharged to home	5,676 (44.9)	887 (46.6)	
Hospital disposition, n (%)			
Died	59 (0.5)	≤ 5	<0.001 [a]
Home without additional supports	9,573 (75.7)	1,500 (78.7)	
Home with additional supports	1,562 (12.4)	216 (11.3)	
Inpatient rehabilitation	545 (4.3)	49 (2.6)	
Nursing home	412 (3.3)	51 (2.7)	
Other	276 (2.2)	57 (3.0)	
Length of stay, median (IQR)	1 (1 – 5)	1 (1 – 4)	<0.001 [b]
Per person healthcare costs, $C, median (IQR)			
Initial care episode	8,070 (2,662 – 15,894)	7,500 (2,527 – 14,238)	<0.001 [b]
30-day	9,532 (4,181 – 18,506)	8,549 (3,709 – 16,252)	<0.001 [b]

[a] Compared using the Chi-square test
[b] Compared using the Wilcoxon-Mann Whitney test

ED = Emergency department

© 2024 Tillmann BW et al. *JAMA Network Open.*

eAppendix 5. Comparison of Characteristics and Outcomes Between Transferred Patients Who Were and Were Not Admitted to a Trauma Center

eTable 11. Baseline Characteristics Stratified by Admission Status

	Admitted (n=6,976)	Discharged from the ED (n=5,676)	SD
Patient Characteristics			
Age, mean (±SD)	49.6 (20.9)	48.0 (21.2)	0.08
Age ≥ 65, n (%)	1,797 (25.8)	1,381 (24.3)	0.03
Female, n (%)	2,615 (37.5)	2,094 (36.9)	0.01
Comorbidity level, n (%)			
Low	3,861 (55.3)	2,924 (51.5)	0.08
Moderate	1,925 (27.6)	1,596 (28.1)	0.01
High	1,190 (17.1)	1,156 (20.4)	0.08
Frail, n (%)	416 (6.0)	372 (6.6)	0.02
Chronic homecare, n (%)	285 (4.1)	215 (3.8)	0.02
Nursing home, n (%)	183 (2.6)	123 (2.2)	0.03
Rural, n (%)	2,314 (33.2)	1,982 (34.9)	0.04
Marginalization score, mean (±SD)	3.0 (0.7)	2.9 (0.8)	0.07
Regular primary care provider, n (%)	6,406 (91.8)	5,264 (92.7)	0.03
ISS, median (IQR)	4 (4 – 9)	4 (1 – 4)	0.90
ISS, n (%)			
<9	5,062 (72.6)	4,421 (77.9)	0.12
9 – 15	1,780 (25.5)	305 (5.4)	0.58
Unable to calculate	134 (1.9)	950 (16.7)	0.53
Mechanism of injury, n (%)			
GSW	59 (0.8)	59 (1.0)	0.02
Cut/pierce	388 (5.6)	700 (12.3)	0.24
Fall	3,292 (47.2)	2,267 (39.9)	0.15
MVC	1,371 (19.7)	706 (12.4)	0.20
Pedestrian/cyclist struck	347 (5.0)	203 (3.6)	0.07
Other blunt mechanism	1,519 (21.8)	1,741 (30.7)	0.20
Triage score, n (%)			
1 (highest acuity)	496 (7.1)	166 (2.9)	0.19
2	3,325 (47.7)	1,945 (34.3)	0.27
3	2,690 (38.6)	2,671 (47.1)	0.17
4	425 (6.1)	832 (14.7)	0.28
5 (lowest acuity)	12 (0.2)	48 (0.8)	0.09
ED arrival density, mean (±SD)	5.0 (3.2)	4.9 (3.2)	0.02
Time of hospital presentation, n (%)			
Weekday	2,529 (36.3)	2,109 (37.2)	0.02
Evening or weekend	3,559 (51.0)	2,809 (49.5)	0.03
Night	888 (12.7)	758 (13.4)	0.02
No. of interhospital transfers, n (%)			
1	6,712 (96.2)	5,404 (95.2)	0.05
≥2	264 (3.8)	272 (4.8)	
Mode of first interhospital transfer, n (%)			
Municipal EMS	4,670 (66.9)	4,406 (77.6)	0.24

© 2024 Tillmann BW et al. *JAMA Network Open.*

	Admitted (n=6,976)	Discharged from the ED (n=5,676)	SD
Critical care transport	2,306 (33.1)	1,270 (22.4)	
Transferring Physician Characteristics			
Sex, n (%)			
Female	1,363 (19.5)	1,474 (26.0)	0.15
Male	3,459 (49.6)	2,604 (45.9)	0.07
Missing	2,154 (30.9)	1,598 (28.2)	0.06
Training, n (%)			
Family medicine residency	2,564 (36.8)	2,298 (40.5)	0.08
Family medicine residency with EM fellowship	1,686 (24.2)	1,420 (25.0)	0.02
Five–year EM residency	463 (6.6)	320 (5.6)	0.04
Missing	2,263 (32.4)	1,638 (28.9)	0.08
Years in practice, n (%)			
<5	1,198 (17.2)	987 (17.4)	0.01
5 – 9	856 (12.3)	728 (12.8)	0.02
≥10	2,756 (39.5)	2,351 (41.4)	0.04
Missing	2,166 (31.0)	1,610 (28.4)	0.06
Non–Trauma Center Characteristics			
Type of hospital, n (%)			
Teaching	1,030 (14.8)	983 (17.3)	0.07
Community	4,219 (60.5)	2,626 (46.3)	0.29
Small	1,719 (24.6)	2,047 (36.1)	0.25
Number of hospital beds, n (%)			
<25	1,420 (20.4)	1,372 (24.2)	0.09
25 – 49	1,493 (21.4)	1,810 (31.9)	0.24
50 – 99	1,197 (17.2)	497 (8.8)	0.25
100 – 199	907 (13.0)	457 (8.1)	0.16
200 – 299	875 (12.5)	671 (11.8)	0.02
≥300	1,084 (15.5)	869 (15.3)	0.01
CT scanner, n (%)	5,162 (74.0)	3,523 (62.1)	0.26
General surgeon, n (%)	5,510 (79.0)	4,142 (73.0)	0.14
Orthopedic surgeon, n (%)	4,601 (66.0)	3,826 (67.4)	0.03
ICU, n (%)	5,332 (76.4)	4,134 (72.8)	0.08
ICU beds, n (%) [a]			
1 – 4	1,308 (24.5)	1,740 (42.1)	0.38
5 – 9	1,326 (24.9)	424 (10.3)	0.39
10 – 19	1,208 (22.7)	860 (20.8)	0.04
20 – 29	939 (17.6)	694 (16.8)	0.02
≥30	551 (10.3)	416 (10.1)	0.01
ED staffing mix, n (%)			
Family physicians	1,404 (20.1)	809 (14.3)	0.16
Family physicians with extra EM training	308 (4.4)	189 (3.3)	0.06
Combination of family physician types	1,253 (18.0)	786 (13.8)	0.11
Mixed	3,968 (56.9)	3,863 (68.1)	0.23
Unknown	43 (0.6)	29 (0.5)	0.01

[a] Limited to the patients who presented to an NTC with an ICU

© 2024 Tillmann BW et al. *JAMA Network Open.*

eTable 12. Hospital Outcomes Stratified by Admission Status

	Admitted (n=6,976)	Discharged from the ED (n=5,676)	p-value*
ED disposition, n (%)			
Admitted	6,979 (100)	0	<0.001 [a]
Discharged to home	0	5,676 (100)	
Hospital disposition, n (%)			
Died	59 (0.9)	0	<0.001 [a]
Home without additional supports	4,403 (63.1)	5,170 (91.1)	
Home with additional supports	1,532 (22.0)	30 (0.5)	
Inpatient rehabilitation	545 (7.8)	0	
Nursing home	250 (3.6)	162 (2.9)	
Other	187 (2.7)	89 (1.6)	
Length of stay, median (IQR)	4 (2 – 9)	0	<0.001 [b]
Per person healthcare costs, $C, median (IQR)			
Initial care episode	13,647 (8,908 – 22,686)	2,471 (1,917 – 3,961)	<0.001 [b]
30-day	15,519 (9,926 – 25,880)	3,812 (2,510 – 6,992)	<0.001 [b]

[a] Compared using the Chi-square test
[b] Compared using the Wilcoxon-Mann Whitney test

ED = Emergency department

© 2024 Tillmann BW et al. *JAMA Network Open.*

eAppendix 6. Impact of Transfer of Patients With Minor Injuries to Trauma Centers on Sector-Specific Healthcare Costs

eTable 13. Impact of Transfer of Patients With Minor Injuries to Trauma Centers on Sector Specific-Healthcare Costs Overall and Stratified by Trauma Center ED Disposition

	All Patients, RR (95% CI)	Trauma Center ED Disposition, RR (95% CI)	
		Admitted	Discharged
Transfer costs	5.92 (5.57 – 6.29)	6.62 (6.14 – 7.13)	3.84 (3.54 – 4.16)
ED care	1.53 (1.51 – 1.55)	1.77 (1.74 – 1.79)	1.21 (1.19 – 1.24)
Acute inpatient care	0.71 (0.69 – 0.73)	1.22 (1.19 – 1.26)	0.09 (0.08 – 0.10)
Physician services	1.01 (0.99 – 1.03)	1.47 (1.44 – 1.50)	0.46 (0.44 – 0.47)
Inpatient rehabilitation and complex continuing care	0.70 (0.63 – 0.78)	1.23 (1.10 – 1.38)	0.07 (0.04 – 0.11)
Outpatient care	1.21 (1.17 – 1.25)	1.11 (1.07 – 1.15)	1.32 (1.25 – 1.39)
Homecare	0.85 (0.80 – 0.90)	1.15 (1.07 – 1.23)	0.52 (0.47 – 0.58)
Nursing home care	0.98 (0.83 – 1.16)	1.11 (0.90 – 1.36)	0.97 (0.73 – 1.30)
Medications and devices	0.88 (0.77 – 1.02)	0.91 (0.76 – 1.08)	1.00 (0.81 – 1.23)
Other costs	0.90 (0.76 – 1.06)	1.22 (1.00 – 1.50)	0.56 (0.43 – 0.72)

ED = Emergency department; RR = Relative ratio

© 2024 Tillmann BW et al. *JAMA Network Open.*

eAppendix 7. Sensitivity Analyses

eTable 14. Impact of Transfer of Patients With Minor Injuries to Trauma Centers on Healthcare Costs Assuming Either the Least or Most Expensive Form of Transport Was Used for Every Interfacility Transfer

	Cases, $C (95% CI)	Matched Controls, $C (95% CI)	Relative Risk (95% CI)
Used least expensive transport method			
Primary cohort	11,357 (11,161 – 11,556)	12,411 (12,278 – 12,545)	0.92 (0.90 – 0.93)
Cases restricted to those admitted to a trauma center	16,875 (16,600 – 17,155)	12,365 (12,201 – 12,531)	1.36 (1.34 – 1.39)
Cases restricted to those not admitted to a trauma center	4,460 (4,343 – 4,580)	11,899 (11,717 – 12,084)	0.37 (0.36 – 0.39)
Used most expensive transport method			
Primary cohort	16,214 (15,955 – 16,476)	12,991 (12,850 – 13,134)	1.25 (1.22 – 1.27)
Cases restricted to those admitted to a trauma center	22,915 (22,557 – 23,279)	12,954 (12,779 – 13,132)	1.77 (1.73 – 1.81)
Cases restricted to those not admitted to a trauma center	7,849 (7,643 – 8,061)	12,561 (12,366 – 12,760)	0.62 (0.61 – 0.64)

© 2024 Tillmann BW et al. *JAMA Network Open.*

eTable 15. Impact of Transfer of Patients With Minor Injuries to Trauma Centers on Healthcare Costs Using Alternative Models: 1) Inverse Probability Weighted Negative Binomial Regression; 2) Use of Multiple Imputation to Estimate Missing Variables for Creation of the Propensity Score; and 3) Exclusion of All Encounters With Missing Physician Characteristics for Creation of the Propensity Score

	Cases, $C (95% CI)	Matched Controls, $C (95% CI)	Relative Risk (95% CI)
IPW model [a]			
Primary cohort	13,516 (13,239 – 13,799)	12,359 (12,109 – 12,615)	1.09 (1.09 – 1.10)
Cases restricted to those admitted to a trauma center	20,044 (19,657 – 20,439)	13,561 (13,307 – 13,819)	1.48 (1.47 – 1.49)
Cases restricted to those not admitted to a trauma center	7,233 (7,090 – 7,380)	14,302 (14,027 – 14,583)	0.51 (0.50 – 0.51)
Multiple imputation			
Primary cohort	13,431 (13,267 – 13,596)	12,781 (12,417 – 13,156)	1.05 (1.03 – 1.07)
Cases restricted to those admitted to a trauma center	19,452 (19,180 – 19,728)	12,689 (12,268 – 13,124)	1.54 (1.50 – 1.56)
Cases restricted to those not admitted to a trauma center	5,964 (5,864 – 6,066)	12,360 (11,894 – 12,864)	0.48 (0.47 – 0.49)
Excluding physician characteristics			
Primary cohort	13,587 (13,364 – 13,813)	12,682 (12,547 – 12,818)	1.07 (1.05 – 1.09)
Cases restricted to those admitted to a trauma center	19,620 (19,311 – 19,932)	12,639 (12,472 – 12,808)	1.55 (1.52 – 1.58)
Cases restricted to those not admitted to a trauma center	5,985 (5,830 – 6,144)	12,280 (12,091 – 12,473)	0.49 (0.47 – 0.50)

IPW = inverse probability weighted

[a] The same model as the primary analysis was used to create the propensity score for the IPW model. The IPW model was then adjusted for age, sex comorbidity, frailty, homecare, nursing home residence, geographic location, socioeconomic status, ISS, mechanism of injury, triage acuity, date, and time.

© 2024 Tillmann BW et al. *JAMA Network Open.*
