# Supplementary material for: Costs of Transfer From Nontrauma to Trauma Centers Among Patients With Minor Injuries
Source: JAMA Netw Open. 2024 Sep 20;7(9):e2434172. doi: 10.1001/jamanetworkopen.2024.34172 (PMC11415792; doi:10.1001/jamanetworkopen.2024.34172)
Supplement: Supplement 2. — Data Sharing Statement [file jamanetwopen-e2434172-s002.pdf]

Data Sharing Statement

Tillmann. Costs of Transfer From Nontrauma to Trauma Centers Among Patients With Minor Injuries. *JAMA Netw Open*. Published September 20, 2024.
doi:10.1001/jamanetworkopen.2024.34172

Data
Data available: No

Additional Information
Explanation for why data not available: The dataset from this study is held securely in coded form at ICES. While legal data sharing agreements between ICES and data providers (e.g., healthcare organizations and government) prohibit ICES from making the dataset publicly available, access may be granted to those who meet pre-specified criteria for confidential access, available at www.ices.on.ca/DAS (email: das@ ices.on.ca).
